# Transmission of multi-level time-bin encoded cluster states over 29 km of partially deployed optical fiber

**DOI:** 10.1038/s41377-026-02465-5

**Published:** 2026-10-01

**Authors:** Philip Rübeling, Robert Johanning, Jan Heine, Oleksandr V. Marchukov, Michael Kues

**Affiliations:** 1 Institute of Photonics, Leibniz University Hannover, Hannover, Germany; 2 PhoenixD Cluster of Excellence, Leibniz University Hannover, Hannover, Germany

**Keywords:** Quantum optics, Single photons and quantum effects, Ultrafast photonics

## Abstract

The next generation of telecommunication networks will rely on the distribution of complex quantum resources to enable secure and transformative information processing, utilizing entanglement and superposition. Cluster states—multi-qubit entangled states that retain entanglement under local measurements—are the central resource for measurement-based quantum information processing and compelling for quantum networking applications including blind quantum computing and quantum key agreement. However, transmitting cluster states over optical fiber has remained elusive with previous approaches, as losses scale exponentially with the number of photons. Here, we report the first transmission of a four-qubit cluster state to two parties over a partially deployed single-mode fiber link. The qubits of this state are encoded in two photons with multi-level time-bins. We directly generate the cluster state by exploiting coherent control of a parametric generation process, rendering a resource-intensive controlled-phase gate obsolete. To enable processing of this state, we introduce beam splitting for time-bins via chirped pulse modulation—a novel, reconfigurable Fourier-domain pulse shaping technique. We certify genuine multi-qubit entanglement and demonstrate one-way computing primitives on the transmitted cluster state. Our approach achieves the transmission of cluster states over optical fiber, establishing complex quantum resources fully compatible with telecommunication infrastructure.

## Abstract (web summary)

We transmit cluster states over 29.5 km of partially deployed optical fiber using multi-level time-bin encoding, demonstrating fiber-compatible complex quantum resources for quantum networking.

## Graphical abstract

**Figure:**
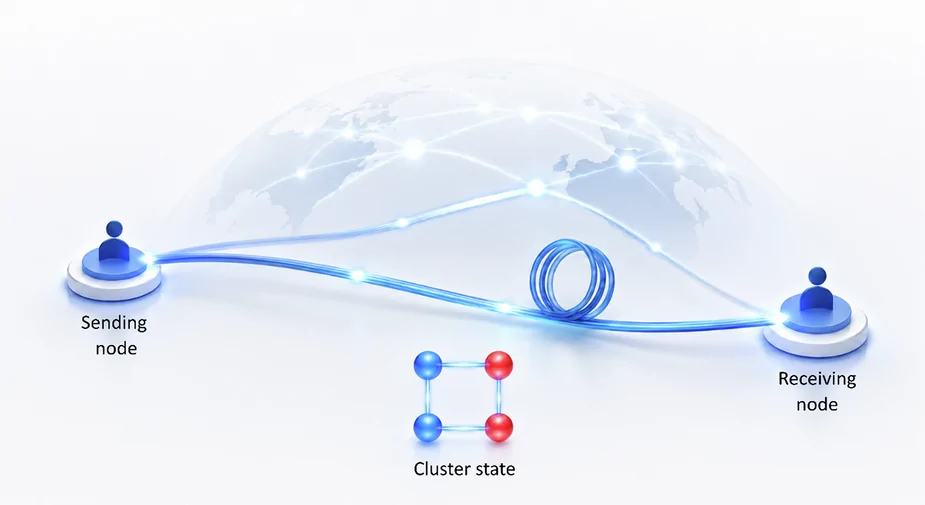

## Introduction

Photonic quantum technologies^1^ utilize superposition and entanglement of light to drive the next revolution in information technology, promising unprecedented possibilities in computing^2^ and secure communications^3^. Quantum networks constitute the backbone of these technologies^4,5^, interconnecting remote users to transfer quantum states as a resource of entanglement^6,7^. Notably, the existing telecommunication infrastructure can be harnessed to implement quantum networks via optical single-mode fiber which paves the way to robust quantum state transfer. At present, deployed fiber networks are being used to distribute entangled photons over hundreds of kilometers^8^, enabling applications such as quantum key distribution^9^. In recent years, multi-qubit entangled states in quantum networks have gained attention, revealing unparalleled capabilities for secure communication and anonymous data processing. In particular, cluster states—multi-qubit quantum states that remain entangled when measured locally—provide a vital resource for advanced quantum networking applications such as distributed quantum computing^10,11^ and blind photonic quantum computing^12,13^.

However, a critical bottleneck to quantum networking is the transmission of complex quantum states (e.g. cluster states) over optical single-mode fiber^14^. Specifically, multi-photon encodings where one qubit is encoded per photon—e.g., polarization encoding^15^—are subject to optical losses that scale exponentially with both photon number and transmission distance. In the absence of loss-tolerant photonic architectures that can overcome this scaling, such as all-photonic quantum repeaters^16^, direct fiber transmission of multi-qubit quantum states encoded across multiple photons remains impractical over application-relevant distances.

An alternative to multi-photon encoding is multi-level encoding, in which multiple qubits are encoded into a single photon, thereby avoiding the exponential loss scaling associated with increasing photon number. Various degrees of freedom have been utilized to implement multi-level encodings. Spatial degrees of freedom, such as orbital angular momentum^17^, are inherently incompatible with single-mode fibers and thus cannot be transmitted over existing telecommunications infrastructure. In contrast, encodings in the time and frequency domain^18^ are natively compatible with optical fibers and today’s telecommunication infrastructure^19–21^ and can be integrated on a photonic chip^22–24^. While cluster states have already been generated in the time and frequency domain, these implementations rely on resource-intensive experimental setups^25^ and continuous active stabilization in multiple degrees of freedom^26^. As a result, their transmission is beyond reach. To transmit cluster states, more robust approaches are required, retaining the high information capacity of the time and frequency domain.

Here, we demonstrate the transmission (i.e., propagation along physically distinct paths) of a four-qubit cluster state over 29.5 kilometers of partially deployed optical single-mode fiber; fully compatible with the existing telecommunications infrastructure. We encoded the cluster state into multi-level time-bin entangled photon pairs and directly generated this state without a controlled-phase gate, substantially reducing the experimental overhead. Moreover, we demonstrate one-way computing primitives on the transmitted cluster state, which is a prerequisite for advanced quantum network applications, such as blind photonic quantum computing. To achieve this, we developed a reconfigurable Fourier-domain pulse shaping technique for time-bin entangled photons, based on highly dispersive CFBGs and electro-optic phase modulation to implement a tunable time-bin beam splitter.

## Results

**Fig. 1:**
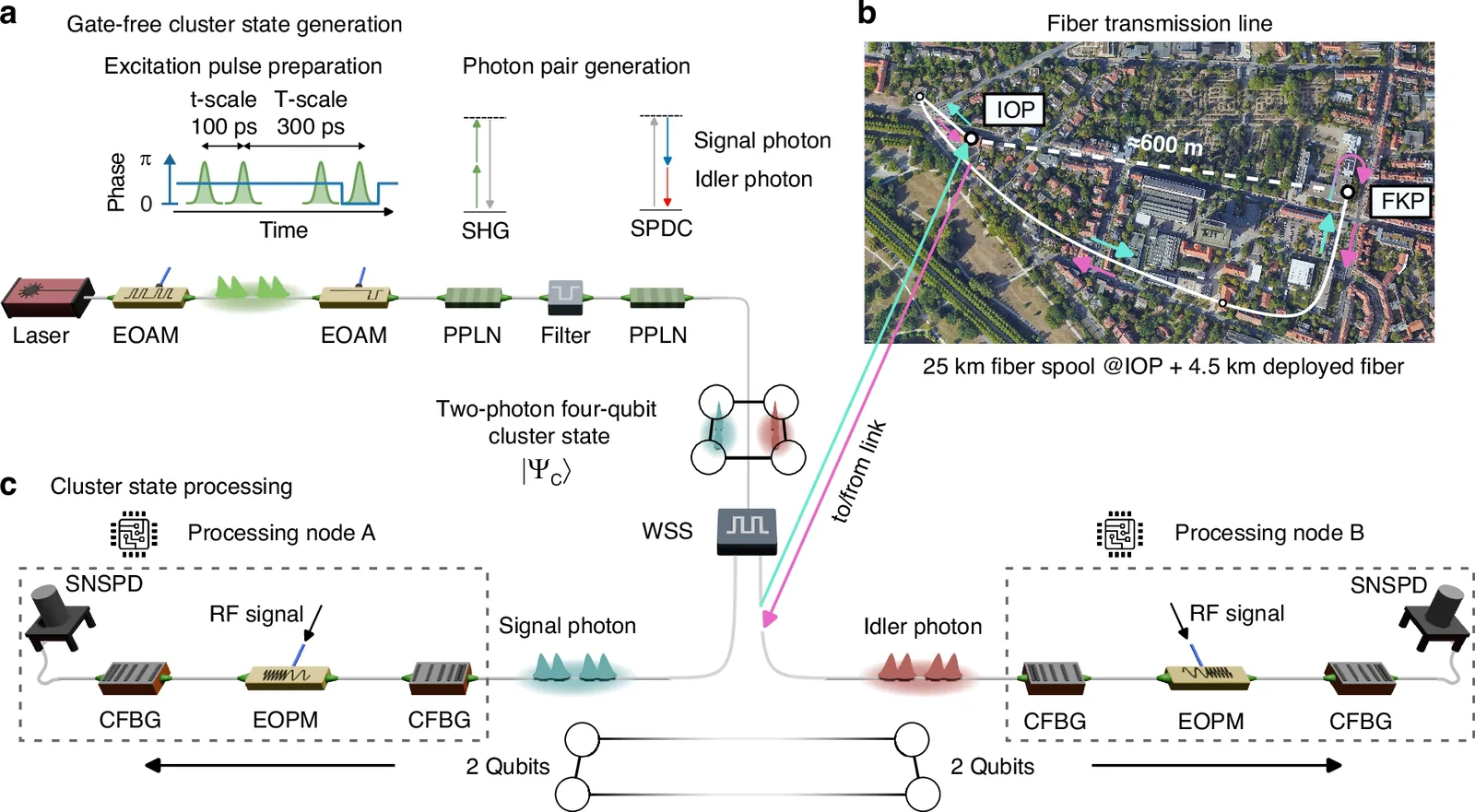
**Schematic of the two-photon four-qubit cluster state distribution experiment.** **a** Gate-free generation of the cluster state $| {\Psi }_{{{\rm{C}}}}\left.\right\rangle$ was realized by carving four coherent excitation pulses with irregular spacings (green) from a continuous-wave laser using an electro-optic amplitude modulator (EOAM). The phase of the fourth pulse was shifted by *π*/2 via an electro-optic phase modulator (EOPM). Time-bin entangled photon pairs were generated using cascaded second-harmonic generation (SHG) and spontaneous parametric down-conversion (SPDC) in a periodically poled lithium niobate waveguide (PPLN). The four qubits of the cluster state are encoded into the photon pair, with each photon carrying two qubits. To transmit the cluster state, signal and idler photons were spatially separated by a wavelength-selective switch (WSS). **b** The idler photon was transmitted over a fiber link on the campus of Leibniz University Hannover connecting the Institute of Photonics (IOP) and the Institute of Solid State Physics (FKP). The linear distance between the institutes was 600 m, while the total round-trip fiber length was 4.5 km. The photons first traveled from the IOP laboratory to FKP (cyan arrows) and were then returned to IOP via a second fiber link (magenta arrows). In addition to this campus link, an extra 25 km of fiber was inserted into the idler path at IOP. **c** Processing and analysis of the cluster state are performed independently for signal and idler photons in two identical setups (Processing Node A for signal photon and Processing Node B for idler photon). In this way, two qubits of the cluster state are transmitted to each processing node. Processing is implemented using chirped pulse modulation in each arm, comprising an electro-optic phase modulator (EOPM) placed between two chirped fiber Bragg gratings (CFBGs). The photons are detected using two superconducting nanowire single-photon detectors (SNSPDs)

In our experiment, we generated time-bin entangled signal-idler photon pairs by cascading second-harmonic generation (SHG) and spontaneous parametric down-conversion excited with four coherent pulses at a telecommunication wavelength. This pulse train was repeated at a repetition rate of 400 MHz. To encode the cluster state into this photonic state, we chose two timescales for the four excitation pulses—one with 100 ps spacing (called *t*-scale) and one with 300 ps spacing (called *T*-scale), see Fig. 1a. In previous work, a controlled-phase gate was used to transform hyper-entangled states into cluster states^26,27^, introducing optical loss to the quantum state and adding experimental complexity. In our approach, a controlled-phase gate is not required. Instead, we utilized an electro-optic phase modulator (EOPM) to apply a phase of *π*/2 to the fourth excitation pulse. Thereby, the quantum state generated via the cascaded parametric processes is not hyper-entangled, but directly the cluster state, as all time-bins of the quantum state have the appropriate phases already applied (see “Experimental setup”). This approach shifts the phase manipulation from the photon pairs to the coherent excitation pulses, reducing optical loss for the quantum state and experimental complexity—critical aspects for quantum network deployments.

We encoded the four-qubit cluster state into four time-bins of the signal and idler photon, respectively. Each photon encodes two qubits: the basis states of the first qubit (called *T*-level qubit) are defined by grouping the first two and the last two time-bins, see Fig. 3a, while for the second qubit (called *t*-level qubit), the basis states are defined by grouping the first and third time-bins and the second and fourth time-bins, see Fig. 3c. Following this encoding, the four-qubit cluster state can be written as

1 $$\begin{array}{rcl}| {\Psi }_{{{\rm{C}}}}\left.\right\rangle &=&\frac{1}{2}\left(| {0}_{T,s}{0}_{T,i}{0}_{t,s}{0}_{t,i}\left.\right\rangle +| {0}_{T,s}{0}_{T,i}{1}_{t,s}{1}_{t,i}\left.\right\rangle \right.\\ &&\left.+| {1}_{T,s}{1}_{T,i}{0}_{t,s}{0}_{t,i}\left.\right\rangle -| {1}_{T,s}{1}_{T,i}{1}_{t,s}{1}_{t,i}\left.\right\rangle \right)\end{array}$$

where each basis state is labeled by signal or idler photon (*s*, *i*) and with respect to the two timescales (*T*, *t*).

Subsequent to the generation of the cluster state, signal and idler photons were de-multiplexed by a wavelength-selective switch and routed into separate fiber paths, selecting 15 GHz channel bandwidth (the pulses had a bandwidth of 11.9 GHz FWHM, corresponding to a Fourier-limited pulse duration of 37 ps FWHM) centered at 193.7 THz (signal photon) and 193.1 THz (idler photon), respectively. This configuration distributed the cluster state into two distinct paths (two qubits each), enabling us to send two qubits of the cluster state to different processing nodes, as illustrated in Fig. 1c. The idler photon was transmitted through a 4.5 km deployed fiber link in a round-trip configuration across the Leibniz University Hannover campus, see Fig. 1b and subsequently sent through an additional 25 km-fiber spool. The signal photon remained in the laboratory. A dispersion compensation module was inserted into the fiber path of the idler photon to compensate for the dispersion introduced by the fiber link and the 25 km fiber spool. Thermal drifts of the fiber length introduced variations on the order of 100 ps in the idler photon arrival time, which can render the detected time-bins indistinct, especially for long integration periods. To compensate for this, we actively stabilized the fiber length with a motorized optical delay line by applying feedback from the time-of-flight of the transmitted idler photon at 15-minute intervals, reducing the jitter to 2.8 ps (FWHM), see “Fiber link stabilization”.

**Fig. 2:**
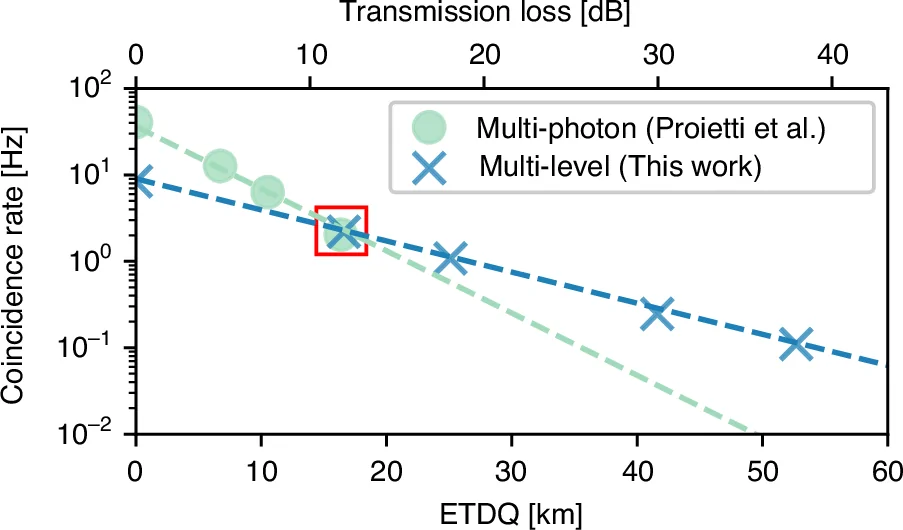
**Coincidence rates for four-qubit states using multi-level encoding (blue) and multi-photon encoding (green) as a function of the effective transmission distance per qubit (ETDQ), defined as the sum of the distances traveled by all qubits divided by the total number of qubits (see SI Appendix D1).** The transmitted states are four-qubit cluster states (this work) and four-qubit GHZ states (Proietti et al.^15^), respectively. Solid curves are obtained from a theoretical rate model (see SI Appendix D2), while dots and crosses indicate experimentally measured values. The red box indicates the experimental condition where the fiber link (29.5 km) was used, without additional attenuation

First, this partially deployed fiber link, together with additional attenuation, was used to probe the distance scaling of the transmission rate (see SI Appendix D1). To transmit a four-qubit quantum state, multi-photon encoding is limited by the need to post-select multi-photon detection events, rendering it more susceptible to optical loss, whereas multi-level encoding requires only two-photon post-selection. Figure 2 compares our four-qubit multi-level encoded state with a four-qubit multi-photon encoded state^15^. The measured data agree well with the rate model (see SI Appendix D2) and reveal a different scaling for multi-level encoding, enabling longer transmission distances.

Next, we certified the presence of genuine multi-qubit entanglement of the transmitted cluster state. To this end, we employed an entanglement witness^28^—derived from the stabilizer group of the respective state—which is an established method to verify multi-qubit entanglement in complex quantum states (see “ Stabilizers and witness”). To compute this witness for the cluster state, local measurements on each qubit need to be performed in two mutually unbiased bases^27^. In our case, this translates to projective measurements in the *Z*-basis and in the *X*-basis that need to be performed on the *t*-level qubits and the *T*-level qubits.

Typically, projective measurements on time-bin encoded qubits are implemented by imbalanced fiber interferometers^29^. However, these interferometers have a fixed imbalance to match a single time-bin spacing, limiting their reconfigurability. Additionally, time-bins can only be interfered in one temporal direction, limiting each measurement setting to a single accessible superposition with only 50% efficiency, i.e. only half of the state amplitude is contributing to the superposition. Moreover, for our multi-level time-bin encoding, four imbalanced fiber interferometers, each stabilized to a reference laser, would be required (for each level for signal and idler, respectively) to obtain the required projective measurement, adding significant experimental complexity.

**Fig. 3:**
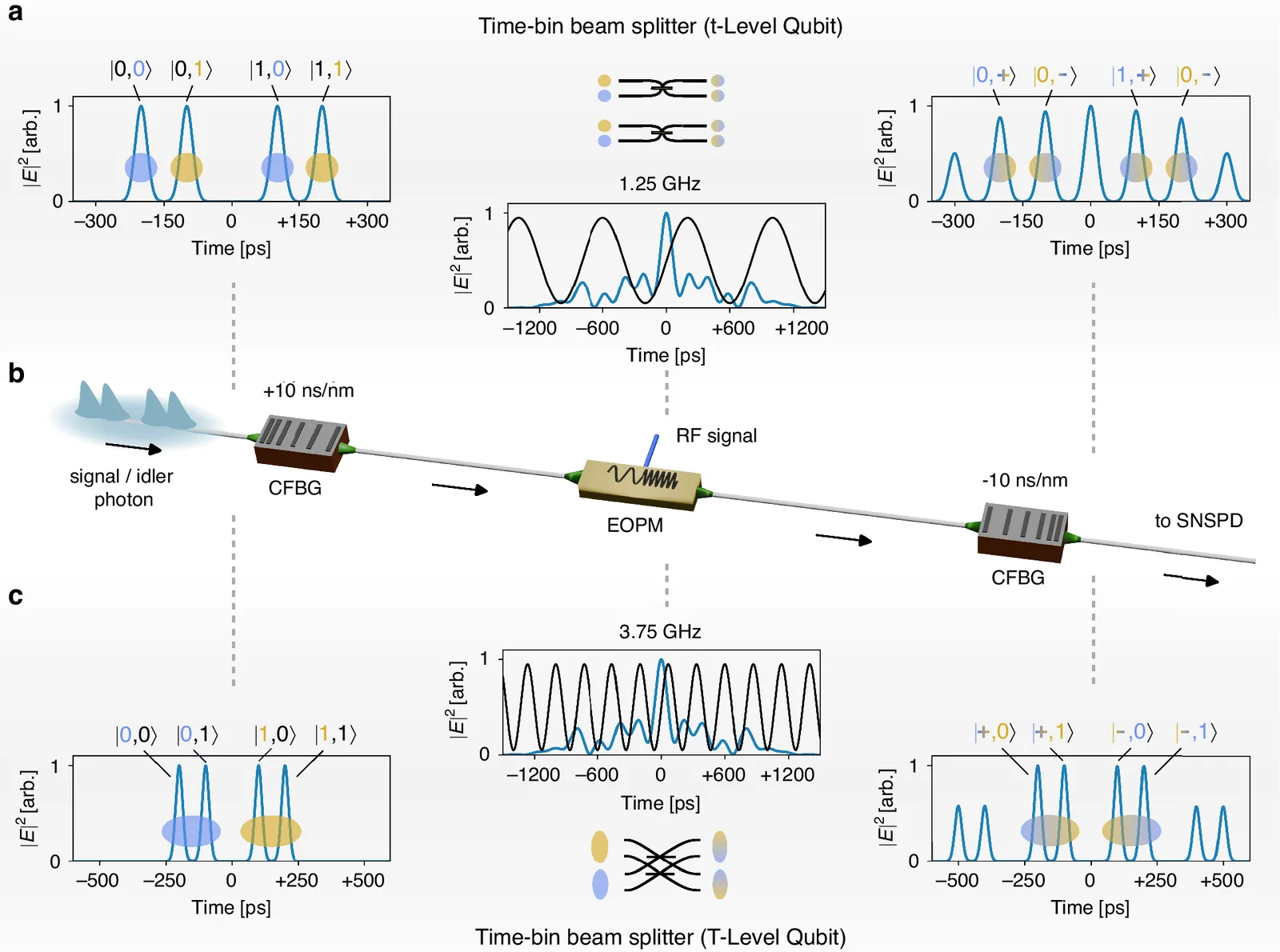
**Reconfigurable time-bin beam splitter operations on multi-level encoded qubits**. **a** The basis states of the *t*-level qubit ($| 0\left.\right\rangle$ in purple, $| 1\left.\right\rangle$ in orange) are encoded into four time-bins of the incoming signal or idler photon (left inset). To superimpose these basis states, a radio-frequency signal at *ω*/(2*π*) = 1.25 GHz (center inset, black curve) is applied to the dispersed pulse (middle inset, blue curve). This yields the superposition states $| +\left.\right\rangle$ and $| -\left.\right\rangle$ of the *t*-level qubit after dispersive recompression, while leaving the *T*-level qubit unaffected (right inset). This implements a time-bin beam splitter acting on the *t*-level qubit. As illustrated in the diagram, the beam splitter operates within the basis states of the *T*-level qubit, hence, two beam splitters are shown. **b** Schematic of the chirped pulse modulation setup, consisting of an electro-optic phase modulator (EOPM) placed between two oppositely chirped fiber Bragg gratings (CFBGs). Rapid changes of the driving signal’s frequency and/or phase enable full reconfiguration of the time-bin beam splitter within a temporal window of  < 2.5 ns. **c** Analogously to the operations on the *t*-level qubit, a radio-frequency signal at *ω*/(2*π*) = 3.75 GHz (center inset, black curve) is applied to superimpose the basis states of the *T*-level qubit (left inset), while leaving the *t*-level qubit unaffected. This implements a time-bin beam splitter acting on the *T*-level qubit. As illustrated in the diagram, the beam splitter operates within the basis states of the *t*-level qubit

To overcome these technological limitations, we introduce chirped pulse modulation (CPM); a novel Fourier-domain wave shaping technique for robust and reconfigurable processing of ultrafast time-bin quantum states, extending the repertoire of Fourier-domain shaping techniques for temporally structured light^30–32^. Here, our experimental setup includes an EOPM located in-between two highly dispersive chirped fiber Bragg gratings (CFBGs) of opposite dispersion. As shown in Fig. 3b, CPM processes time-bins in three sequential steps: (i) The CFBG with positive chirp (+10 ns/nm) maps the spectrum of the time-bins to the time domain, approximating a dispersive Fourier-transform^33,34^ (see “Chirped pulse modulation”). (ii) The spectral phase is modulated by an EOPM on which a radio-frequency waveform is applied. (iii) The CFBG with negative chirp (−10 ns/nm) maps the spectral components back into the time domain, approximating an inverse dispersive Fourier-transform. As a result, coherent copies of the time-bins are generated at integer multiples of *Δt* = ∣*β*_2_∣*ω*, where *β*_2_ is the group velocity dispersion of the CFBGs, and *ω* is the angular frequency of the radio-frequency waveform (see “Chirped pulse modulation”). Importantly, we controlled the phase of the radio-frequency signal with respect to the arrival time of the time-bins and adjusted the signal amplitude, implementing a reconfigurable time-bin beam splitter. Our time-bin beam splitter is based on post-selection and can generate superposition states with an intrinsic efficiency (i.e., ignoring the insertion loss) of 60% (see SI Appendix G1).

To perform CPM on our time-bin encoded cluster state, we drove the EOPM with an arbitrary waveform generator (AWG) at radio-frequencies of 1.25 GHz to superimpose time-bins of 100 ps (*t*-level qubits), see Fig. 3a and of 3.75 GHz to superimpose time-bins of 300 ps (*T*-level qubits) spacing, see Fig. 3c. To ensure phase synchronization across the setup, a single AWG was used to drive the modulators for the excitation pulse shaping and the CPM. We applied a single segmented waveform at the AWG, realizing nine different beam splitter settings. For the first setting, no modulation was applied, resulting in a projective measurement in the *Z*_*T*_*Z*_*T*_*Z*_*t*_*Z*_*t*_-basis (computational basis). The next four beam splitter settings facilitate coherent superpositions of the *t*-level qubits, which are realized with two different beam splitter phases (0, *π*/4) for signal and idler photons, respectively, resulting in projective measurements in the *Z*_*T*_*Z*_*T*_*X*_*t*_*X*_*t*_-basis. The last four beam splitter settings facilitate coherent superpositions of the *T*-level qubits, again with the same phase settings for the beam splitter, resulting in projective measurements in the *X*_*T*_*X*_*T*_*Z*_*t*_*Z*_*t*_-basis. We normalized the projective measurements for each basis to compensate for the different modulation settings (see SI Appendix E). The waveform was repeated every 180 ns, allowing all nine settings to be measured in parallel, making our approach robust to fluctuations in the count rates, caused e.g. by polarization rotations and pump power fluctuations.

**Fig. 4:**
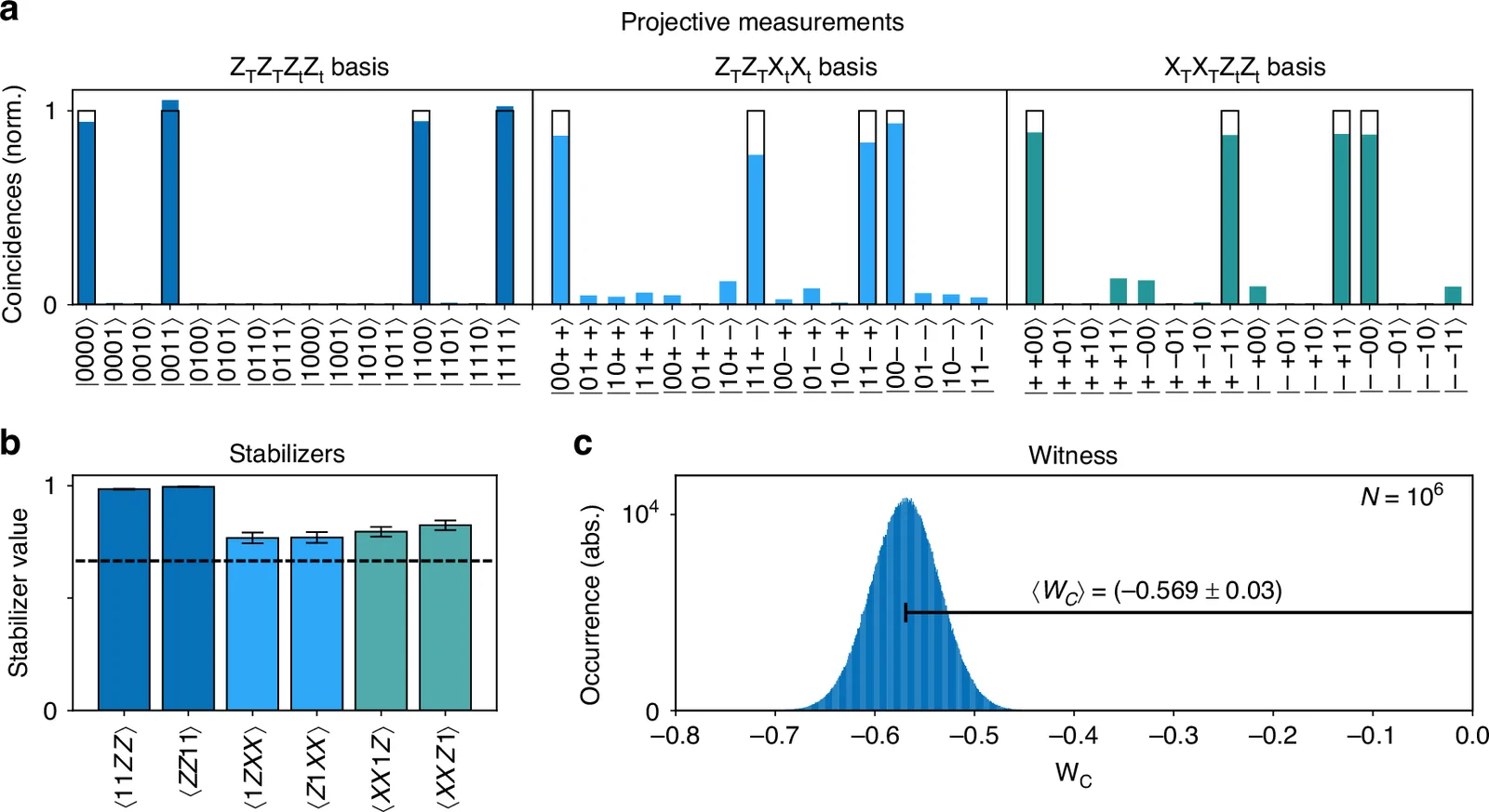
**Certification of the cluster state after distribution over 29.5 km of fiber. a** The 48 projective measurements in three different basis settings are shown, i.e., *Z*_*T*_*Z*_*T*_*Z*_*t*_*Z*_*t*_-basis (measurements in the computational basis), *Z*_*T*_*Z*_*T*_*X*_*t*_*X*_*t*_-basis (superpositions of the *t*-scale qubits) and *X*_*T*_*X*_*T*_*Z*_*t*_*Z*_*t*_-basis (superpositions of the *T*-scale qubits) (see “Chirped pulse modulation”). The projections are normalized with respect to the total coincidence counts in the corresponding measurement setting (i.e., *ZZZZ*, *ZZXX*, *XXZZ*). Black boxes indicate expected values for the ideal cluster state. **b** Stabilizer values of the four-qubit cluster state. The black dashed line indicates the threshold value of 2/3 that all stabilizers need to exceed to verify multi-qubit entanglement. **c** The witness *W*_*C*_ = ( − 0.57 ± 0.03) is obtained from the stabilizers. The uncertainty of 19 standard deviations was estimated by a Monte Carlo simulation. The histogram shows the (Gaussian) distribution of the witness values for 10^6^ samples of the Monte Carlo simulation (see “Stabilizers and witness”)

From the nine configurations of the time-bin beam splitter, we extracted 48 projective measurements, see Fig. 4a, which we used to compute the stabilizers of the cluster state, see Fig. 4b. From the stabilizers, we calculated the witness *W*_*C*_ = (−0.57 ± 0.03), verifying the presence of multi-qubit entanglement after transmission through optical fiber with a confidence of 19 standard deviations, see Fig. 4c.

**Fig. 5:**
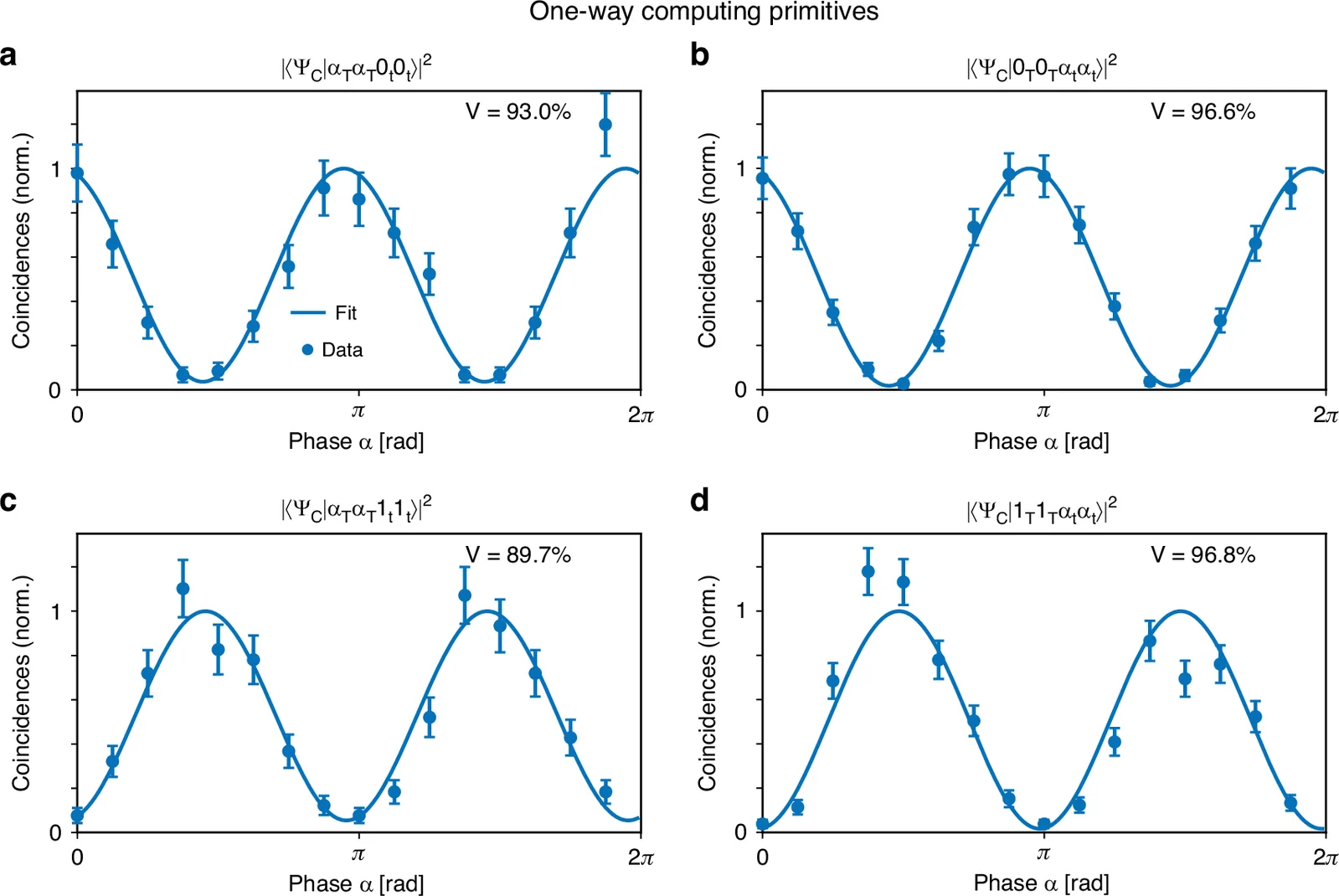
**One-way computing primitives after transmission over 29.5 km of fiber**. We perform local measurements on different subsets of qubits of the transmitted cluster state. Two qubits were measured in a basis rotated in the *XY*-plane of the Bloch sphere (with phase *α*), and the two remaining qubits were measured in the *Z*-basis (see SI Appendix E). By fitting the raw data (no background subtraction) to the expected interference pattern, we achieved visibilities of **a** 93.0 % for the projection onto $| {\alpha }_{T,s},{\alpha }_{T,i},{0}_{t,s},{0}_{t,i}\left.\right\rangle$, **b** 96.6 % for the projection onto $| {0}_{T,s},{0}_{T,i},{\alpha }_{t,s},{\alpha }_{t,i}\left.\right\rangle$, **c** 89.7 % for the projection onto $| {\alpha }_{T,s},{\alpha }_{T,i},{1}_{t,s},{1}_{t,i}\left.\right\rangle$ and **d** 96.8 % for the projection onto $| {1}_{T,s},{1}_{T,i},{\alpha }_{t,s},{\alpha }_{t,i}\left.\right\rangle$, all above the CHSH-threshold of 70.7 %. The visibilities correspond to *S*-parameters of **a** *S* = 2.63, **b** *S* = 2.73, **c** *S* = 2.54, and **d** *S* = 2.74^36^

To showcase the applicability of the transmitted cluster state for quantum networking applications, we performed primitives of one-way computing operations on a subset of qubits^35^. To accomplish this, we measured two-qubit quantum interference on the cluster state by projecting the *t*-level qubits into one of the *Z*-basis states, while interfering the *T*-level qubits and vice versa, see Fig. 5. The visibilities of all measurements exceed 89.7%, clearly violating the CHSH inequality^36^, proving the residual bipartite entanglement after partial projective measurements on the transmitted cluster state.

## Discussion

In this work, we have demonstrated the transmission of a cluster state over partially deployed single-mode fiber. Our results show, for the first time, that cluster states can be transmitted over fiber. This was achieved using multi-level encoding, which circumvents the loss burden of multi-photon encoding. Our demonstration utilized a resource-efficient scheme for generating two-photon cluster states and chirped-pulse modulation (CPM) for manipulating time-bins across different timescales. The transmitted state was validated by a cluster state witness and by one-way computing primitives.

In our experiment, the witness value and visibilities are upper-bounded, resulting from imperfections of the time-bin beam splitter (see SI Appendix B). First, it does not realize a clean two-mode operation and therefore introduces cross-talk. This can be improved by cascading CPMs and EOPMs and by using multi-tone modulation (see SIAppendix G2). Second, its visibility is reduced by spectral walk-off (see “ Chirped pulse modulation”). This could be overcome by operating in a different regime, with larger dispersion, reduced jitter, and shorter pulse durations and separations (see SI Appendix C). Our proof-of-principle demonstration establishes the transmission of intermediate-size complex quantum resources, while not constituting a directly scalable fault-tolerant architecture.

The distribution topology of our approach is restricted to two nodes. However, this is sufficient for the target applications, such as blind quantum computing^12,37^ and quantum key agreement^38^. In the present experiment, both nodes were located in the same laboratory, larger spatial separation could be achieved using synchronization via radio-frequency over optical fiber^39,40^. In our experiment, the main limitation on transmission distance is optical loss, primarily in the CPM setup. The loss could be alleviated by utilizing integrated components such as electro-optic phase modulators^41^ and arrayed waveguide gratings^42^, tailoring the source phase-matching bandwidth to the pulse bandwidth to enhance the pair generation rate, and exploiting the full fiber bandwidth through spectral multiplexing (see SI Appendix F).

Importantly, the rate model analysis of the multi-photon encoding compared to the multi-level encodings performed in this work (see  SI Appendix F), does not encompass fault-tolerant architectures (e.g., those proposed in refs. ^16, 43–46^), where redundancy could compensate for photon loss. In the present comparison of loss-susceptible encodings, loss of one photon removes all information carried.

Here, we were able to demonstrate one-way computing primitives; however, multi-qubit operations and feed-forward could also be implemented with our approach (see SIAppendix D1) and enable distributed universal one-way quantum computing. While it is challenging to scale to large cluster states due to the exponentially growing mode space, 10 qubits could already be achievable with our approach using state-of-the-art technology (see SI Appendix C). Distributed cluster states of this size could open up new applications for quantum networks based on one-way computing, such as a distributed blind Grover algorithm^47^ and multi-qubit quantum communication^38^.

## Materials and methods

### Experimental setup

To prepare coherent excitation pulses, we used a continuous-wave laser (Koheras Adjustik HP15, NKT Photonics) centered at 193.4 THz. Its output field was modulated with an electro-optic amplitude modulator (IM-1550-40-V-PM, Optilab) driven by an AWG (M8194A, 120 GSa/s, Keysight). The amplitude modulator was used to shape a train of four excitation pulses, where each pulse had a duration of 37 ps (full-width at half maximum). We used an irregular temporal spacing for the pulses; 100 ps between the first and second pulse, 200 ps between the second and third pulse, and 100 ps between the third and fourth pulse. The entire pulse sequence was repeated four times every 10 ns, corresponding to 400 MHz repetition rate. To ensure stable operation of the amplitude modulator, we split 50% of the signal to a bias controller (ABC, ID Photonics).

After creating the pulse pattern, we applied a phase of 0 to the first three pulses and a phase of *π*/2 to the fourth pulse with an electro-optic phase modulator (EOSpace Inc.), which was driven by the same AWG. Next, we used an erbium-doped fiber amplifier (Lumibird Inc.) to increase the average optical power to 5 mW. We launched this pulse train into a periodically poled lithium niobate waveguide (PPLN, Covesion Ltd.) with 40 GHz phase-matching bandwidth to perform SHG, converting the excitation pulses at 193.4 THz (1550 nm) to 386.8 THz (775 nm). In this process, two fundamental photons were converted into a single photon at the second-harmonic frequency. For the phase-shifted fourth excitation pulse, this process doubles the previous applied phase to *π*. Subsequently, we filtered the fundamental (193.4 THz) and passed the 775 nm light through a PPLN to directly generate the two-photon cluster state via spontaneous parametric down-conversion (SPDC).

To spectrally filter the signal and idler photons of the created cluster state and to route them to individual fibers, we used a programmable filter (Waveshaper 4000A, Finisar) and selected an optical bandwidth of 15 GHz each. The signal photon remained inside the lab, while the idler photon was transmitted over a 4.5 km deployed fiber link in a round-trip configuration with 5.3 dB insertion loss and subsequently transmitted over 25 km of commercial low-loss single-mode fiber (SMF-28 Ultra, Corning Inc.) with 5.3 dB insertion loss. This fiber was wound on a spool and thermally isolated in a polystyrene box, experiencing temperature fluctuations of <0.1 K. Assuming a thermal sensitivity of 36.8 ps/(K ⋅ km)^48^, the resulting time-of-flight jitter of the spool is estimated to be 92 ps. The time-of-flight jitter of the fiber link was on the order of 100 ps. We reduced the total time-of-flight jitter to 2.8 ps (FWHM) by active stabilization of the fiber length, see “Fiber link stabilization”. The dispersion of the single-mode fiber was compensated with a dispersion compensating module (insertion loss 2.4 dB, dispersion of −450 ps/nm).

To implement CPM in our experiment, we used CFBGs with a positive dispersion of +9988 ps/nm (CB-P100H-LU01FA, insertion loss 2.2 dB, Proximion AB) and a negative dispersion of −9982 ps/nm (CB-N100H-LU01FA, insertion loss 3 dB, Proximion AB). The spectral phase modulation in the CPM was performed with an electro-optic phase modulator (EOSpace Inc., insertion loss 3.2 dB (idler) and 2.9 dB (signal), electro-optic bandwidth 40 GHz), which was driven by the AWG. We used one CPM setup for the signal and idler photons, each with an insertion loss of ~8.4 dB (idler) and 8.1 dB (signal). Losses of all individual components are listed in Table 1.

**Table 1:** Optical loss budget for signal and idler photons

Component	Loss (idler)	Loss (signal)
Waveshaper	6.5 dB	6.5 dB
Deployed link (4.5 km)	5.3 dB	–
Fiber spool (25 km)	5.3 dB	–
Dispersion compensation	2.4 dB	–
Optical delay line	1.5 dB	–
CFBG (+10 ns/nm)	2.2 dB	2.2 dB
CFBG (−10 ns/nm)	3.0 dB	3.0 dB
EOPM	3.2 dB	2.9 dB
Total	29.4 dB	14.6 dB

*CFBG* chirped fiber Bragg grating, *EOPM* electro-optic modulator

The photons were detected using two superconducting nanowire single-photon detectors (EOS, Single Quantum) connected to a time-to-digital converter (TimeTagger Ultra, Swabian Instruments). The total jitter budget is listed in Table 2. The measured pulse length (FWHM, including jitter) was ~60 ps. An electronic reference signal (rectangular pulse with 10 ns duration, every 180 ns) was forwarded from the AWG to the time-to-digital converter to assign photon arrival times to the segments of the radio-frequency waveform.

**Table 2:** Temporal jitter budget in the experiment

Component	Jitter (FWHM)
Pulse duration (filtered)	49 ps
TDC conversion	19 ps
SNSPD	15 ps
Link	2.8 ps
Electronics	0.7 ps
Total	58 ps

Each jitter is given as the full-width at half-maximum. The overall jitter is calculated under the assumption of independent Gaussian error propagation

### Fiber link stabilization

The optical fiber in the idler path was actively stabilized with a motorized optical delay line (MDL-02, Luna Innovations) to compensate time-of-flight variations. Feedback was applied every 15 min (corresponding to 1 mHz feedback bandwidth) using batches of single-photon detection events accumulated over the preceding interval. For each batch, the (mean) photon arrival time was determined from a Gaussian fit to the single-photon counts in the *ZZZZ*-basis (i.e., without modulation).

**Fig. 6:**
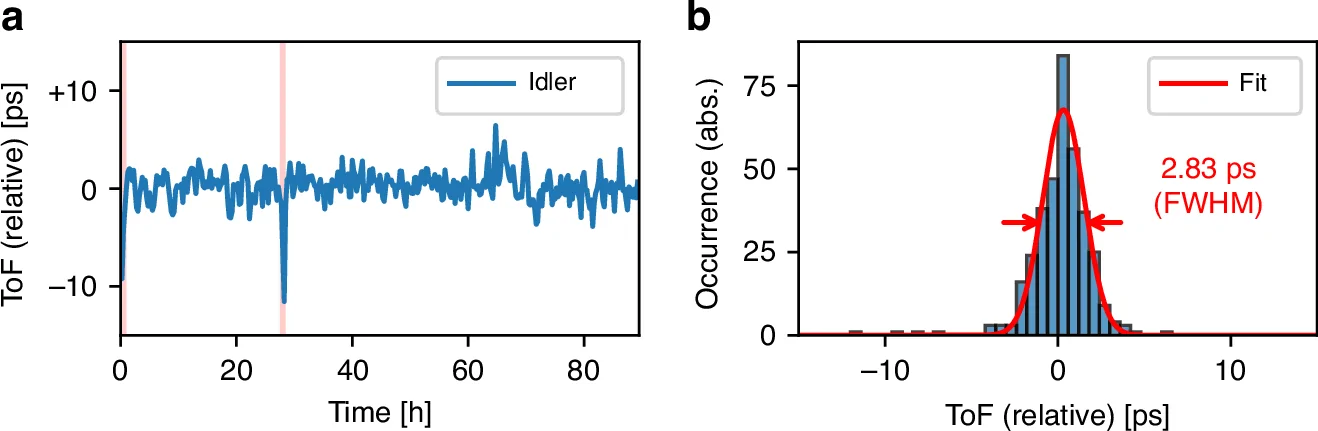
**Time-of-flight (ToF) jitter of the idler photons after transmission through the 4.5 km deployed fiber link and the 25 km fiber spool.** **a** ToF jitter recorded over 89.5 h with 15 min averaging intervals. The initial offset results from a short tracking interruption between the initial phase calibration and the subsequent measurement. **b** Histogram of the ToF. Gaussian fitting (excluding the outlier after 28 h, marked in red) reveals residual link stability of 2.8 ps (FWHM)

Initially, the arrival time was calibrated to a fixed reference delay corresponding to the targeted radio-frequency-to-optical (RFtO) phase. During operation, deviations from this reference were compensated by the delay line. Long-term stability was verified over the full acquisition time of ~90 h, confirming stable time-of-flight operation throughout, as shown in Fig. 6. To stabilize the idler-photon polarization, real-time feedback was implemented with a microcontroller (Arduino Uno) driving stepper motors on the polarization paddles to maximize the count rate.

### Chirped-pulse modulation

**Fig. 7:**
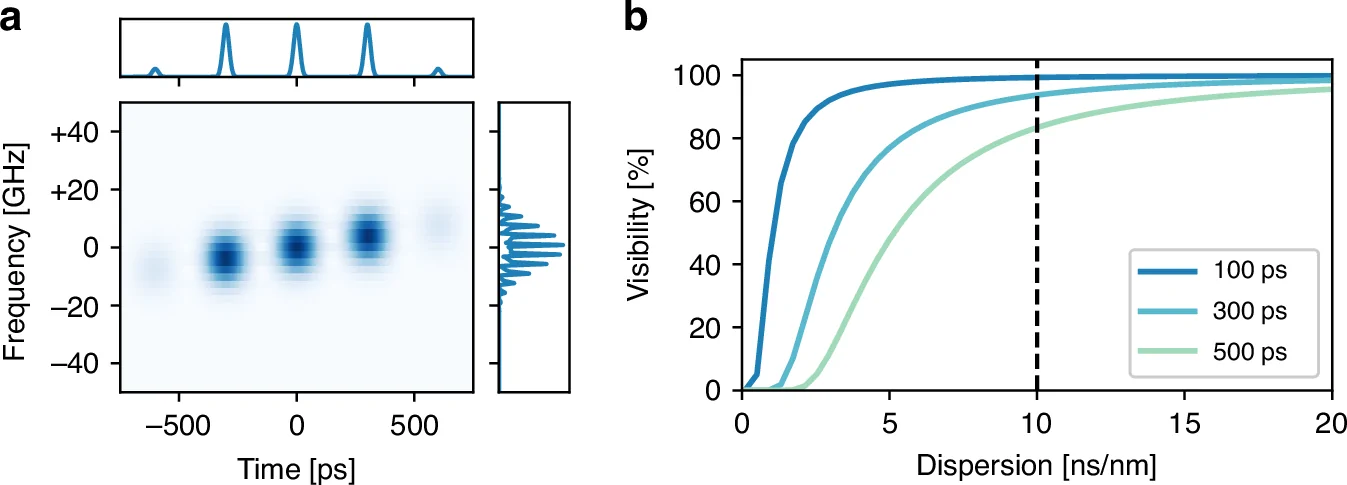
**Spectral walk-off and quantum interference visibility bounds of chirped pulse modulation.** **a** Time-frequency spectrogram (simulated) for a single Gaussian input pulse with 37 ps pulse duration (FWHM) after being processed by chirped pulse modulation (*ω*/(2*π*) = 3.75 GHz and 10 ns/nm dispersion). The center frequencies of the pulse copies at ±300 ps (and ±600 ps) are spectrally shifted with respect to the original input pulse at 0 ps. This spectral walk-off diminishes the mutual overlap of interfering time-bins, bounding the interference visibility. **b** Bounds on the quantum interference visibility for two time-bins at three temporal spacings (100 ps, 300 ps, 500 ps) as a function of the dispersion of the CFBGs. The dashed black line indicates the dispersion value of the CFBGs used in this work (±10 ns/nm). At this dispersion value, the quantum interference visibility bounds are given by 99.3% for 100 ps, 94.0% for 300 ps and 84.1% for 500 ps, respectively

CPM applies a dispersive Fourier transformation to temporally separate the spectral components of input pulses, enabling spectral phase modulation. The CPM affects both the temporal and spectral properties of the output pulses. Specifically, by applying a sinusoidal voltage $V(t)={V}_{0}\sin (\omega t+\alpha )$ to the CPM, a time-bin at time *t* and frequency *ν* is converted into coherent pulse copies of different order *m* (*m* = 0, ± 1, ± 2, …), which are shifted in time by *m* ⋅ *Δt* and in frequency by *m* ⋅ *Δν*. Mathematically, this can be described by the transformation

2 $$| \nu ,t\left.\right\rangle \to {\sum }_{m=-\infty }^{\infty }{J}_{m}(g){e}^{-im\alpha }| \nu +m\Delta \nu ,t+m\Delta t\left.\right\rangle$$

where *J*_*m*_ denotes the Bessel functions of the first kind and order *m* and *g* = π *V*_*0*_/*V*_*π*_  is the modulation depth. The time and frequency shifts are given by *Δt* = *β*_2_*ω* and *Δν* = *ω*/(2*π*), respectively, where *β*_2_ is the dispersion of the CFBG. When two time-bins at the same frequency are launched into the CPM, temporally overlapping pulse copies at the output do not have perfect spectral overlap due to their spectral shift, diminishing the interference contrast. As shown in Fig. 7, this effect bounds the visibility to ≤94% for 300 ps time-bin spacing (T-level qubits) and to ≤99.3% for 100 ps spacing (t-level qubits). The phase *α*, which is controlled via relative timing adjustments of the RF waveform with respect to the time-bins, enables precise control over the time-bin interference.

### Stabilizers and witness

The stabilizer formalism provides a framework to quantify entanglement of multi-qubit quantum states^49^, requiring fewer measurement settings compared with, e.g., quantum state tomography^50^. For our approach, we used the stabilizer formalism to construct a witness operator which diagnoses the presence of multi-qubit entanglement within the given quantum state. A negative expectation value of the witness operator certifies the presence of genuine multi-qubit entanglement^51,52^. For the four-qubit cluster state that we consider here, a witness operator is given by refs. ^27,53^

3 $$\begin{array}{rcl}{W}_{C}&=&2-\frac{1}{2}\left(11ZZ+ZZ11+1ZXX\right.\\ &&\left.+Z1XX+XX1Z+XXZ1\right)\end{array}$$

where *X* and *Z* are Pauli operators and 1 is the identity operator. The choice of this witness is not unique, however, it is particularly suitable to our experiment, as it exclusively requires measurement either on the t-level or on the T-level qubits and permits linear incoherent noise of up to 33.3%. Moreover, this type of witness can be generalized to generic multi-qubit and high-dimensional cluster states^54^. We estimated the statistical error of the stabilizers and witness using a Monte Carlo resampling method based on the measured counts. Assuming Poisson statistics, we generated 10^6^ samples to compute witness values for each sample and extracted the standard error from the resulting distribution. Moreover, based on the witness, a lower bound to the fidelity of our state with respect to the ideal cluster state is given by *F* ≥ (1 − *W*_*C*_)/2 = 78.5% ^49^.

## Supplementary information

Supplementary Information for Transmission of multi-level time-bin encoded cluster states over 29 km of partially deployed optical fiber.

## Data Availability

The data that support the findings of this study are available from the corresponding author on reasonable request.
