# Supplementary material for: Transmission of multi-level time-bin encoded cluster states over 29 km of partially deployed optical fiber
Source: Light Sci Appl. 2026 Oct 1;15:384. doi: 10.1038/s41377-026-02465-5 (PMC13627715; doi:10.1038/s41377-026-02465-5)
Supplement: Supplementary file 1 [file 41377_2026_2465_MOESM1_ESM.pdf]

Supplementary Information for

Transmission of multi-level time-bin encoded cluster states over 29 km of partially deployed optical fiber

Philip Rübeling,[1,2,†] Robert Johanning,[1,2,†] Jan Heine,[1,2] Oleksandr V. Marchukov,[1,2] and Michael Kues[1,2,*]

[1] *Institute of Photonics, Leibniz University Hannover, Nienburger Straße 17, 30167 Hannover, Germany*

[2] *PhoenixD Cluster of Excellence, Leibniz University Hannover, Nienburger Straße 17, 30167 Hannover, Germany*

(Dated: August 3, 2026)

[†] These authors contributed equally to this work.

[*] michael.kues@iop.uni-hannover.de

Appendix A: Additional experimental results

In the measurement reported in the main manuscript, we transmitted a cluster state between two CPM modules separated by 29.5 km of optical fiber. In addition, we performed a reference measurement in which the cluster state was not transmitted through fiber but instead measured locally in the laboratory. This yielded a cluster state witness of $W_C = (-0.75 \pm 0.06)$, which we used as a benchmark for the directly generated cluster state. The fidelity of this locally generated cluster state can be lower bounded by $F = (1 - W_C)/2 \geq 87.5\%$. Comparing this value with the fidelity of the transmitted state shows a reduction in fidelity of 9.5% after transmission. The reason for this is imperfect phase calibration of the CPM due to the additional optical losses, see section B. We also carried out an additional experiment in which both photons (signal and idler) of the cluster state were transmitted in common mode through a single 25 km fiber spool. This effectively corresponds to a scenario in which each photon experiences 25 km of transmission. A key difference in this configuration was that only a single chirped pulse modulation setup was used: both photons propagated through the same positively dispersive CFBG, the same electro-optic phase modulator (EOPM), and the same negatively dispersive CFBG. Due to the 600 GHz spectral separation between the signal and idler channels, their arrival times become offset after the first CFBG, even though they enter it simultaneously. By the time they reach the EOPM, the two photons are temporally separated and can therefore be modulated independently using different temporal segments of the same device, see Fig. S1(a). The projective measurements obtained by this scheme are shown in Fig. S1(b). Under these conditions, we obtained a cluster state witness of $W_C = (-0.80 \pm 0.04)$, corresponding to a minimum fidelity of $F = (1 - W_C)/2 \geq 90\%$.

Appendix B: Noise model for the witness

We developed a noise model for the experiment to identify the sources of witness degradation before and after transmission. Therefore, we calculated the contributions of the electro-optic modulation up to the sidebands of third order for all projection measurements.

For the cluster state before transmission (i.e., the reference measurement in Appendix A), the dominant noise is induced by the non-ideal beam splitting operations and arises from scattering of photons into undesired time-bins. This results in cross-talk in the $ZZXX$ basis and bounds the witness to $W_C = -0.877$. The effect could be avoided by utilizing ideal time-bin beam-splitting operations (see Appendix G 2). Further noise is induced by the spectral walk-off, limiting the two-photon interference visibility to 94% for 300 ps (T-level qubits) and to 99.3% for 100 ps (t-level qubits), see Fig. 6. We incorporated this effect in our noise model, which suggests that this causes an additional witness degradation to $W_C = -0.752$, which is in agreement with our experimental result $W_C = (-0.75 \pm 0.06)$.

The dominant error source responsible for the witness and fidelity degradation after the transmission was imperfect phase calibration of the CPM, which was caused by the high optical losses of our testbed. The transmission added 14.8 dB attenuation to the idler photon, significantly reducing the coincidence count rate. As we used a fixed time to calibrate the phases of the CPM for the modulation on the T-scale and the t-scale, for signal and idler, respectively, the phase error increased compared to the reference measurement. For our simulation, we have chosen the maximal phase offset (including the one-sigma confidence interval from the fit) from our one-way computing measurements in Fig. 5. Including this phase error of $\approx 2.7\%$, the simulation of all leading error sources matched with the experimental projections, see Fig. S2. With this additional witness degradation of 0.137, our noise model predicts a witness of $W_C = -0.607$, close to our experimental value of $W_C = -0.569 \pm 0.03$. The phase error can be minimized by reducing optical loss in our experiment, i.e. by using loss-optimized integrated optics and by using deployed fiber links with less than ≈ 1.2 dB/km. Also longer measurement times would improve the phase measurement accuracy.

A further candidate for witness degradation due to the transmission was potential influence of thermal noise arising from fluctuations in both ground and ambient air temperatures during measurements conducted at peak summer conditions. Typical temperature variations of approximately 5–10 K (ground) and 2–5 K (air) introduce phase and amplitude instability in the state. Based on the residual jitter on the link, we expect an absolute timing stability of about 2.8 ps. For the modulation signals, this implies for our system (1.25 GHz = 800 ps) and (3.75 GHz = 267 ps) RFtO phase drifts of approximately $2\pi \cdot (2.8\text{ps}/800\text{ps}) = 0.022$ rad and $2\pi \cdot (2.8\text{ps}/267\text{ps}) = 0.066$ rad, respectively. These contributions are therefore not relevant for the witness degradation with an upper bound of 0.007.

Appendix C: Scalability

Multi-level encoded quantum states are challenging to scale in system size (i.e., the number of qubits), because of the exponentially growing mode space. However, systems of intermediate size (~ 10 qubits) are sufficient to demonstrate and implement new functionalities in quantum networking, significantly exceed the current state of the art in transmission distance and comparable number of qubits [1, 2]. Using currently available technology, our platform could be scaled to encode graph states with up

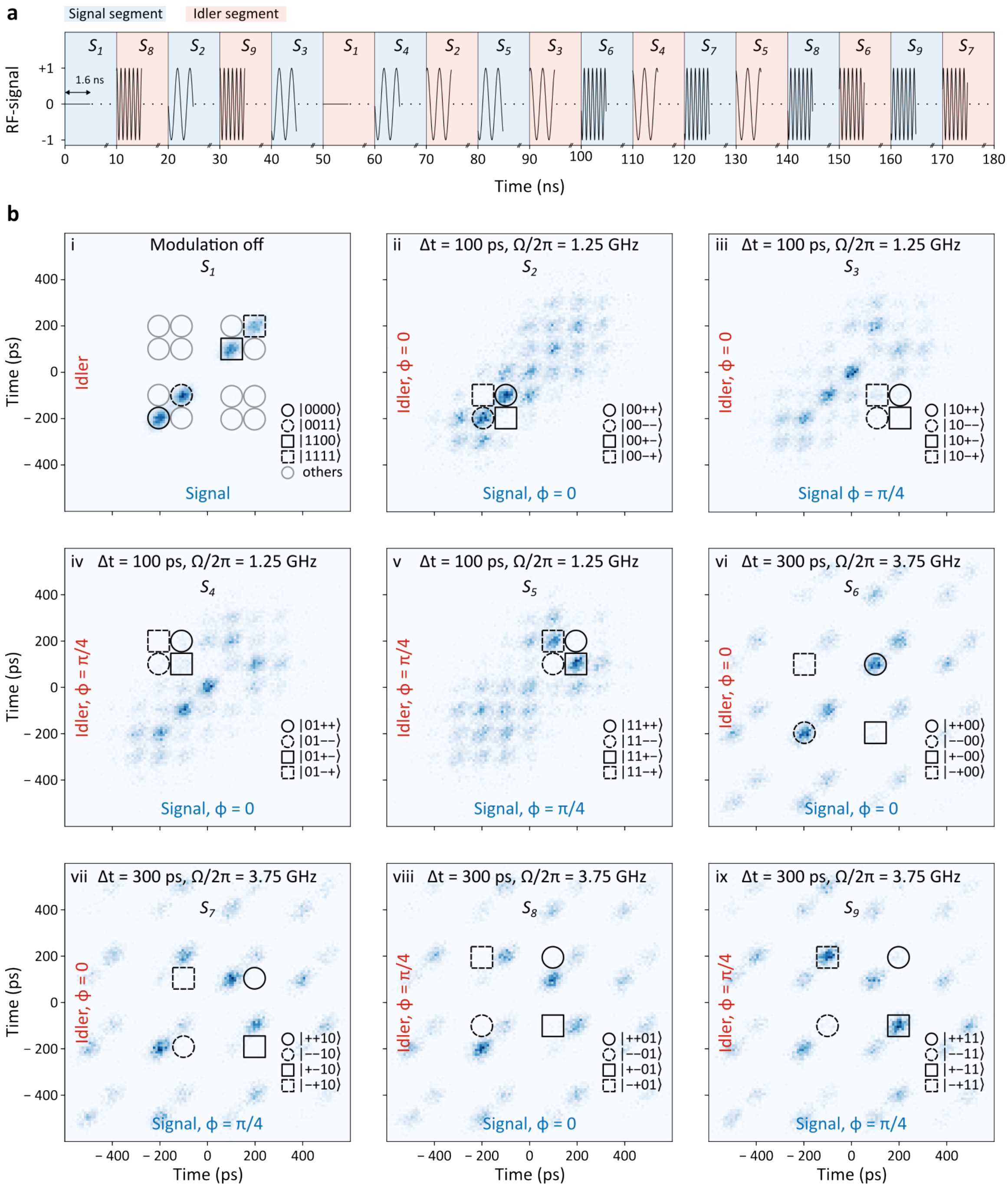

Figure S1. **Radio-frequency waveform used for the chirped pulse modulation for the additional results and corresponding joint temporal intensity measurements for the cluster state.** (a) The radio-frequency waveform applied to the interleaved chirped pulse modulation was composed of 18 segments each 10 ns long (here, only the first 1.6 ns are displayed for each segment). Signal and idler photons were temporally separated by 48 ns after the first chirped fiber Bragg grating, enabling their independent modulation. Red and blue colors indicate whether signal or idler photons were modulated by the respective segment. Two modulation tones (1.25 GHz for modulation at 100 ps on the t-scale and 3.75 GHz for modulation at 300 ps on the T-scale) with different phases relative to the time-bins were used to access all relevant 48 projections necessary to construct the witness operator. (b) Each pair of complementary waveform segments (labeled with Latin letters) generates one out of the nine displayed joint temporal intensity patterns. All 48 projections required for witness reconstruction can be derived from these measurements. Only a subset of projections was takes for each measurement setting, which are indicated by the circles and boxes. The projections in the other bins were not orthogonal.

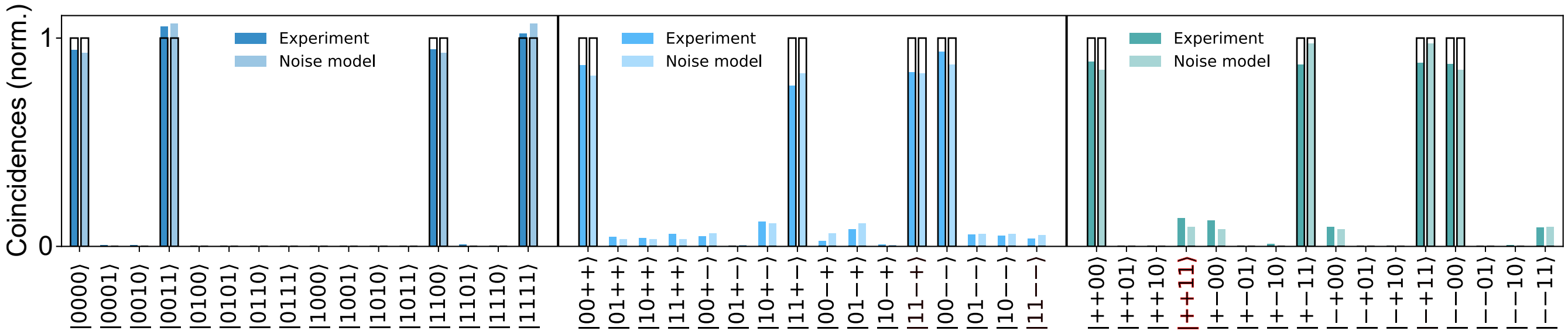

Figure S2. **Simulated projection measurements based on our noise model and comparison with experimentally obtained projection measurements.** The left bars of each pair show our experimental projections after transmission over a 4.5 km deployed fiber link and a 25 km fiber spool (see Fig. 4 (a)). The right bars show the corresponding projections predicted by our noise model. In the ZZZZ-basis (left), the deviation of both experimental and simulated projections from the ideal case (black bars) originates from skewed of the pump pulses; however, this effect has only a minor influence on the witness. In the ZZXX-basis (middle), noise is primarily caused by EOPM scattering noise and phase error due to limited alignment accuracy, while spectral walk-off contributes only weakly. In the XXZZ basis (right), reduced visibility due to spectral walk-off is the dominant noise source, whereas phase errors have only a minor impact in this configuration.

to 10 qubits (i.e., 5 qubits per photon).

1. Formal analysis

Formally, any graph states with $2n$ qubits can be represented in the two-photon multi-level encoding, where each photon encodes n qubits. To prove this, consider a graph state represented by the undirected graph $G = (V, E)$, where V denotes its set of vertices and E denotes its set of edges. The representation of this graph state in the canonical basis is given by

$$|G\rangle = \prod_{(i,j)\in E} \mathrm{CZ}_{ij} |+\rangle^{\otimes 2n}, \tag{S1}$$

where CZ_{ij} denotes the controlled-Z gate acting on the two qubits associated with the edge (i, j) and $|+\rangle = (|0\rangle + |1\rangle)/\sqrt{2}$. For this graph state, consider a bipartition into two subgraphs $G_A = (V_A, E_A)$ and $G_B = (V_B, E_B)$, such that $G = (G_A|G_B)$, with $|V_A| = |V_B| = n$. In this bipartition, the state can be written as

$$|G\rangle = \sum_{s=1}^{r} \eta_s |\psi_s^A\rangle \otimes |\varphi_s^B\rangle, \tag{S2}$$

where η_s are complex numbers, $|\psi_s\rangle \otimes |\varphi_s\rangle$ are orthonormal state vectors in the bipartite basis and r is the Schmidt rank of the state with respect to the bipartition. To transfer the state from the canonical basis representation to the bipartite basis representation, a local unitary transformation $\mathrm{LU}_1 \otimes \ldots \otimes \mathrm{LU}_{2n}$ needs to be applied, where each individual local unitary transformation LU_j is an element of the Clifford group. For the equal bipartition of a quantum state with $2n$ qubits, the Schmidt rank satisfies [3]

$$r \leq \min(2^{|V_A|}, 2^{|V_B|}) = 2^n. \tag{S3}$$

The Schmidt rank of the bipartite representation implies, that any graph state can be written as a two-photon state with at most 2^n basis states (e.g., time-bins) per photon. In Fig. S3 a procedure is shown, which, for a given graph state with $2n$ qubits, provides the mapping of basis state (i.e., the state encoding) and the excitation pulse sequence to generate the graph state.

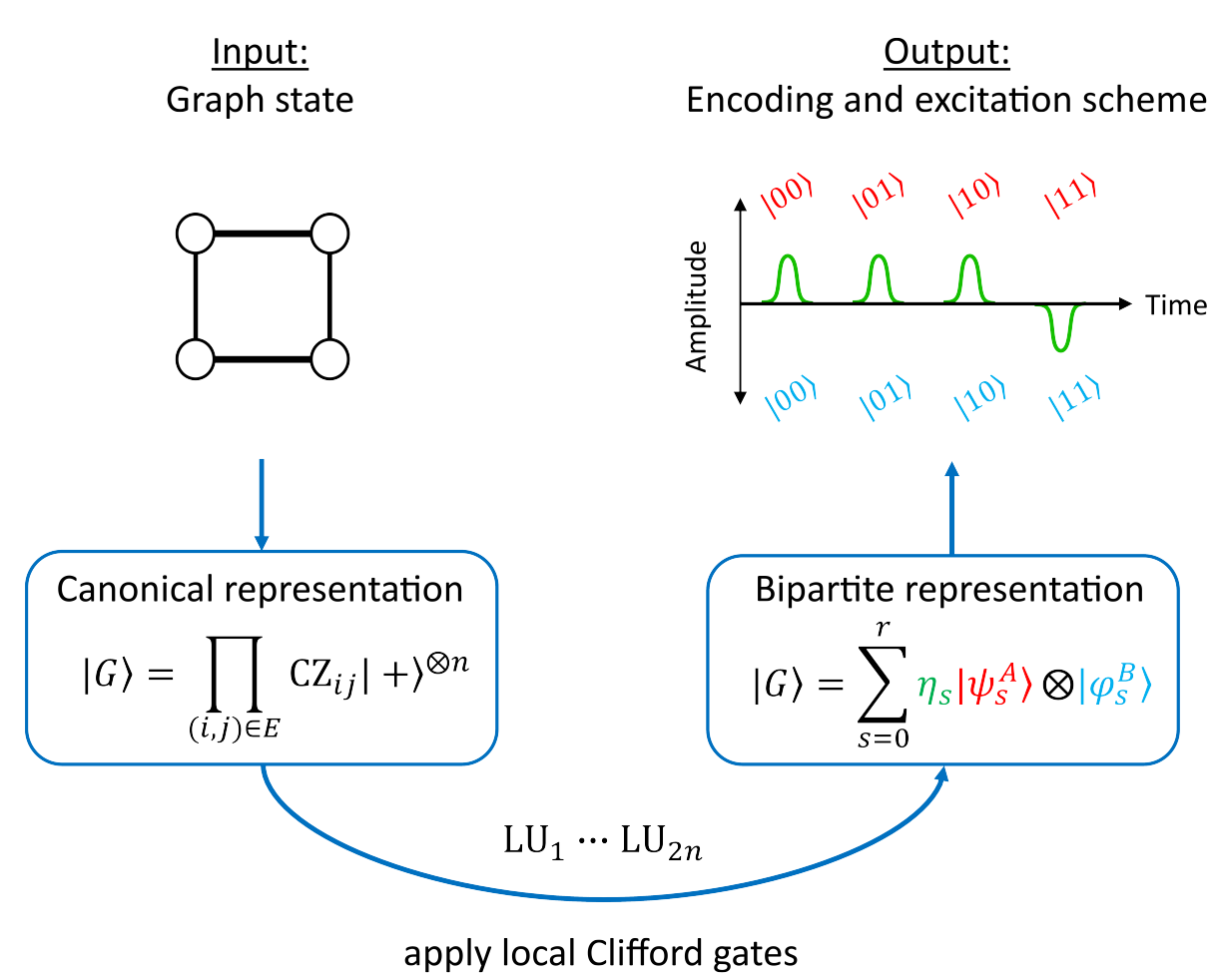

Figure S3. **Graphical representation of the procedure to obtain the encoding and excitation scheme for a graph state.**

To process the n qubits encoded in a photon, n layers of beam-splitters need to be applied per photon. While the specific action of each layer of beam splitters depends on the chosen encoding scheme and can be different for the qubits encoded in the signal and idler photon (i.e.,

the mapping of qubits states and time-bin can be different), the combined unitary transformations can be implemented with n layers for example using the Clements-decomposition [4].

2. Examples for 6-qubit graph states

The 4-qubit cluster state has already been discussed in the manuscript; the next states to consider are 6-qubit cluster states. To illustrate the general scheme outlined in the previous section, the excitation pulse scheme and the encoding of the *star* graph state $|S_6\rangle$, the *dumbbell* graph state $|D_6\rangle$, the *cluster* graph state $|C_6\rangle$, the *triangle* graph state $|T_6\rangle$ and the *butterfly* graph state $|B_6\rangle$ are discussed in this section, see Fig. S4.

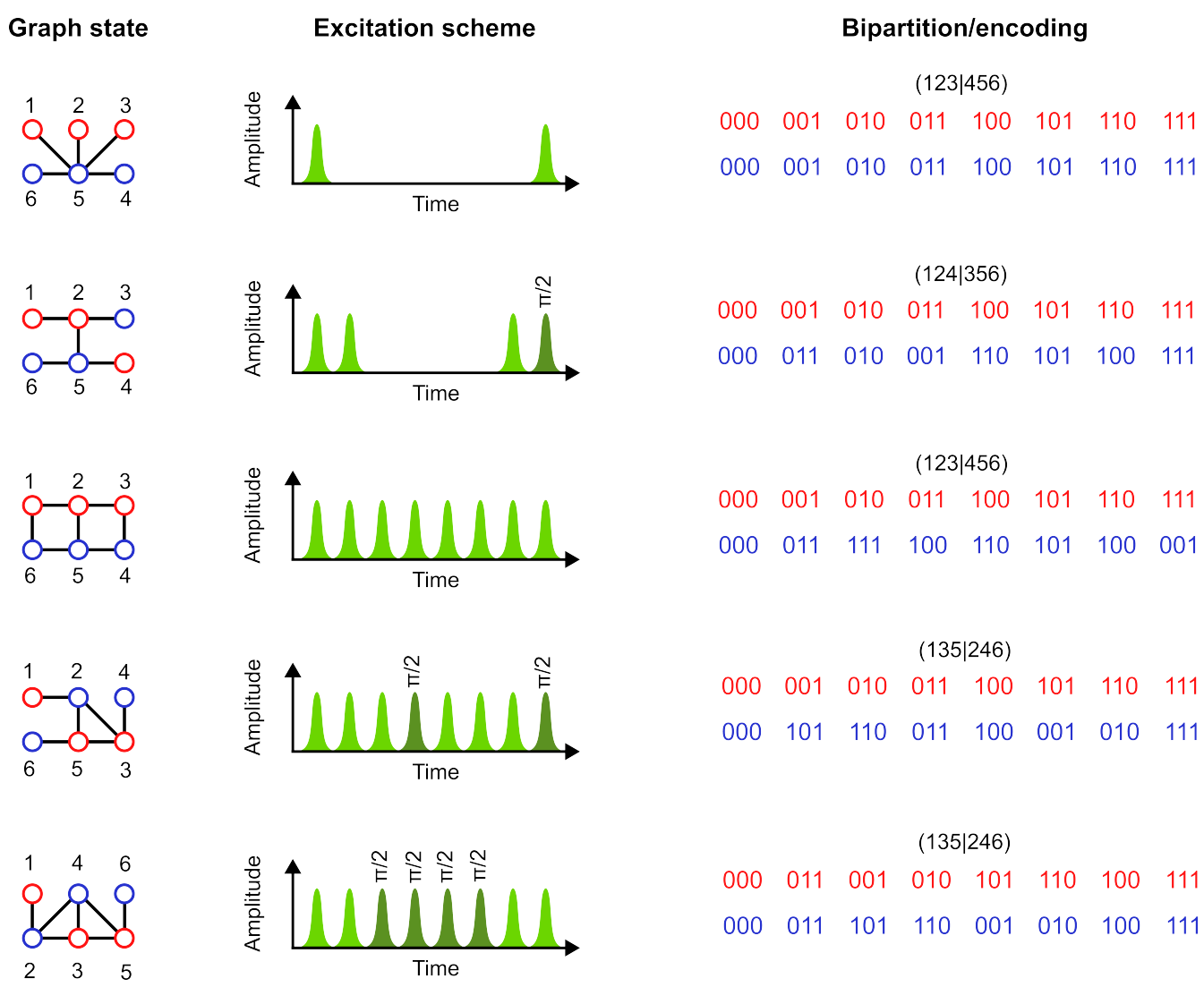

Figure S4. **Excitation scheme and bipartite encoding for 6-Qubit graph states.** Qubits that belong to the idler/signal photon are red/blue.

Following the canonical representation, and applying a local Clifford operation $H \otimes H \otimes H \otimes H \otimes H \otimes 1$, the bipartite representation of the *star* graph state is

$$|S_6\rangle = \frac{1}{\sqrt{2}}\big(|000\rangle|000\rangle + |111\rangle|111\rangle\big), \tag{S4}$$

which means, this state can be represented by two excitation pulses $(+,+)$, see Fig. S4(a). The bipartite representation of the *dumbbell* graph state is obtained by relabeling the qubits according to the bipartition (124|356) and subsequently applying a local Clifford operation $H \otimes 1 \otimes H \otimes H \otimes 1 \otimes H$ to the canonical representation. The state can be written as

$$\begin{aligned}|D_6\rangle =& \frac{1}{\sqrt{4}}\big(|000\rangle|000\rangle + |001\rangle|011\rangle \\ &+ |110\rangle|100\rangle - |111\rangle|111\rangle\big),\end{aligned} \tag{S5}$$

and implemented with a sequence of four excitation pulses $(+,+,+,-)$, see Fig. S4(b). Next, the *cluster* graph state can be constructed by considering the bipartition (123|456) and subsequently applying a local Clifford operations $1 \otimes H \otimes 1 \otimes H \otimes 1 \otimes H$ and is given by

$$\begin{aligned}|C_6\rangle = \big(& + |000\rangle|000\rangle + |001\rangle|011\rangle \\ &+ |010\rangle|111\rangle + |011\rangle|100\rangle \\ &+ |100\rangle|110\rangle + |101\rangle|101\rangle \\ &+ |110\rangle|010\rangle + |111\rangle|001\rangle\big)/\sqrt{8},\end{aligned} \tag{S6}$$

which can be implemented by a sequence of 8 excitation pulses $(+,+,+,+,+,+,+,+)$. Next, the *triangle* graph state can be constructed by considering the bipartition (135—246) together with the local Clifford operation $1 \otimes 1 \otimes H \otimes H \otimes H \otimes 1$ resulting in

$$\begin{aligned}|T_6\rangle = \big(& + |000\rangle|000\rangle + |001\rangle|101\rangle \\ &+ |010\rangle|110\rangle - |011\rangle|011\rangle \\ &+ |100\rangle|100\rangle + |101\rangle|001\rangle \\ &+ |110\rangle|010\rangle - |111\rangle|111\rangle\big)/\sqrt{8},\end{aligned} \tag{S7}$$

which can again be represented by a sequence of 8 excitation pulses $(+,+,+,-,+,+,+,-)$. Last, the *butterfly* graph state can be implemented by the bipartition and by applying the local Clifford operation $1 \otimes HS \otimes 1 \otimes HS \otimes HS \otimes H$. The resulting state can be written as

$$\begin{aligned}|B_6\rangle = \big(& + |000\rangle|000\rangle + |011\rangle|011\rangle \\ &- |001\rangle|101\rangle - |010\rangle|110\rangle \\ &- |101\rangle|001\rangle - |110\rangle|010\rangle \\ &+ |100\rangle|100\rangle + |111\rangle|111\rangle\big)/\sqrt{8},\end{aligned} \tag{S8}$$

and again implemented by considering an excitation pulse sequence with 8 pulses $(+,+,-,-,-,-,+,+)$.

3. Limitations

While the multi-level encoding is scalable to larger resource states, three limitations need to be taken into account. The first limitation arises from the exponentially growing modal space in state generation. A state comprising $2n$ qubits requires 2^n modes per photon. Consequently, the system repetition rate must be reduced, leading to an exponential suppression of the state-generation rate with increasing system size. Although this limitation ultimately is fundamental, it can be partially alleviated by spectral multiplexing. The second limitation arises from the non-ideal operation of the time-bin beam splitter. Scattering into undesired modes degrades the efficiency and fidelity of the resulting transformations. This limitation is technical rather than fundamental and could be mitigated by a

quasi-ideal beam splitter, enabling mutually unbiased multi-qubit operations with negligible crosstalk. The third limitation arises from the spectral walk-off, which reduces the interference visibility and, consequently, the fidelity of the quantum operations. This limitation is technical rather than fundamental and could be mitigated by utilizing time-bins of shorter duration and separation, dispersive components specifically tailored to time-bin bandwidth and cascaded dispersive elements.

The first two limitations do not yet constitute the primary constraint on scalability in the present implementation, the third currently represents the dominant limitation in our experiment. Overcoming it will require a more careful engineering of the system, as outlined in the following section.

4. Hardware requirements

To access multi-level encoding with 32 time-bin modes (i.e., 5 qubits per photon), several design parameters of our system could be improved to alleviate the spectral walk-off, while maintaining the current system performance (i.e., optical insertion loss, visibility and repetition rate).

The first design parameters are the pulse duration and the temporal separation of the time bins, which could be reduced to 5 ps and 20 ps, respectively, by using an actively mode-locked laser as the excitation source at a repetition rate of 50 GHz [5]. This regime is accessible with state-of-the-art equipment, provided that the overall system jitter can be further reduced. This regime provides two advantages. First, owing to their bandwidth of 88.2 GHz (FWHM), the 5 ps pulses are substantially less susceptible to spectral walk-off the and the modulation frequency can be reduced further, as a smaller temporal spacing is sufficient. We suggest a (fundamental) modulation frequency of 50 MHz at a dispersion of 50 nm/ns to address the short time-scale of 20 ps. Our numerical simulation suggests, that to address the largest time-scale of 640 ps required in this regime (modulation frequency of 1.6 GHz), the visibility is given by 98.4 %. For larger separations of 1280 ps the interference still has a visbility of 93.3 %.

To access this regime, the CFBGs must be redesigned. The relevant design trade-off is set by the relation between the acceptance bandwidth B and the dispersion D, which satisfy $B \cdot D = 2cL/n$ where L is the length and n is the (mean) refractive index of the fiber used to implement the grating. In the current implementation, the CFBG fiber length is 8.5 m and the (mean) refractive index is $n = 1.46$, corresponding to an acceptance bandwidth of $B = 1$ THz and a dispersion of $D = 10$ ns/nm. A 5 ps pulse fits well within a $B = 200$ GHz acceptance window, such that, for the same device length and comparable loss, the dispersion could in principle be increased to $D = 50$ ns/nm. In addition, the center wavelengths of the gratings must be matched to the signal and idler, respectively. To scale the number of time-bins beyond 100, multiple cascaded CFBGs need to be considered. This is feasible in principle up to at least 150 ns/nm [6]. The main challenges are the alignment of the acceptance bandwidths and the reduction of losses, for example by directly cascading the fibers in transmission and thereby avoiding the use of a circulator, which presently contributes a substantial part (~ 0.7 dB) of the total insertion losses of the CFBGs.

Appendix D: Quantitative comparison of multi-level and multi-photon encodings

1. Effective Transmission Distance per Qubit (ETDQ)

To enable a quantitative comparison between distinct distributed settings, the notion of transmission distance must be operationalized. A suitable metric should be independent of the quantum-state encoding and the topology of the distributed setting. To this end, we define the effective transmission distance per qubit (ETDQ) as

$$\mathrm{ETDQ} = \frac{\sum_{n\in \mathrm{N}} q_n \cdot l_n}{\sum_{n\in \mathrm{N}} q_n}, \tag{S1}$$

where N is the set of nodes in the setting, q_n is the number of qubits sent to node n, and l_n is the fiber distance (normalized to an attenuation of 0.18 dB/km) from source to node n. For example, ETDQ $= l$ for both a four-node star network with one qubit sent to each node and a two-node line network with two qubits sent to each node, provided each node is at distance l from the source. In the experiment we used different combined setting of the hybrid link and variable attenuators were utilized to emulate different ETDQ. For each setting, we measured the attenuation, we converted it into an equivalent fiber length, see Table 1. The same was done for the different settings considered in [7]. In both cases, we compare the raw coincidence count rates.

2. Rate model

To assess the achievable transmission distances of multi-level and multi-photon encodings, the rate at which a $2n$ qubit quantum state can be distributed over a given ETDQ l is considered. Multi-level and multi-photon encodings are compared under identical conditions and assumptions, including state generation, transmission, and processing. This rate refers to the relevant detection event, namely an $2n$-photon coincidence for multi-photon encoding and a 2-photon coincidence for multi-level encoding. It is obtained by tracing each photon from gen-

Table 1. **Effective Transmission Distance per Qubit (ETDQ) and Coincidence Count Rate (CCR) for different experimental settings.** To convert between attenuation and ETDQ we used the attenuation coefficient of low-loss SMF-28 single-mode fiber (0.18 dB/km).

Configuration	Loss	ETDQ	CCR (raw)
–	0 dB	0 km	8.41 Hz
Link	11.95 dB	16.4 km	2.20 Hz
Link + 6 dB	18.07 dB	25.1 km	1.08 Hz
Link + 18 dB	29.95 dB	41.6 km	0.25 Hz
Link + 26 dB	37.94 dB	52.7 km	0.11 Hz

eration to detection. Formally, the rate can be written as the product of three factors

$$R_{\mathrm{tot}}(n,l) = R_{\mathrm{G}}(n) \cdot R_{\mathrm{T}}(n,l) \cdot R_{\mathrm{P}}(n), \qquad \text{(S2)}$$

where $R_{\mathrm{G}}(n)$ is the generation rate of the $2n$ qubit quantum state, $R_{\mathrm{T}}(n,l)$ is the transmission probability over effective length l and $R_{\mathrm{P}}(n)$ is the transmission through the processing setup, including detection. Table 2 summarizes the rate of each step as a function of the ETDQ l and the number of qubits n, assuming probabilistic photon pair sources and universal linear optics in both cases.

3. Rates for multi-photon encoding

The $2n$-qubit state is generated from n photon pairs, each generated with probability p_{G}. To entangle these pairs requires $(n-1)$ probabilistic fusion measurements, each with success probability $p = 1/2$. This gives an overall generation probability of $R_G(n) = p_{\mathrm{G}}^n/2^{n-1}$. These $2n$ photons are transmitted over an effective distance l, yielding a transmission probability of $R_{\mathrm{T}}(n,l) = \exp(-2n\alpha l)$, where α is the fiber attenuation (here, assumed to be 0.18 dB/km corresponding to $\alpha = 0.958$/km). Finally, all $2n$ photons pass through the state analyzers with transmission p_A and are detected with efficiency p_D, resulting in a probability of $R_{\mathrm{P}}(n) = p_A^{2n} p_D^{2n}$. For the 4-qubit multi-photon encoded state in [7], the probabilities are given by $p_{\mathrm{G}} = 5.65 \cdot 10^{-3}$, $p_{\mathrm{A}} = 0.52$ and $p_{\mathrm{D}} = 0.95$ and the laser repetition rate was $f_{\mathrm{R}} = 320$ MHz.

4. Rates for multi-level encoding

The $2n$-qubit state is directly generated from one photon pair and its mode space requires 2^n modes, The probability of generating a photon-pair in one temporal mode is p_{G}. This gives an overall generation probability of $R_G(n) = p_{\mathrm{G}}/2^n$. These 2 photons are transmitted over an effective distance l, yielding a transmission probability of $R_{\mathrm{T}}(n,l) = \exp(-2\alpha l)$, where α is the fiber attenuation. Finally, both photons pass through n state analyzers with transmission p_A and are detected with efficiency p_D, resulting in a probability of $R_{\mathrm{P}}(n) = p_A^{2n} p_D^2$. For the 4-qubit multi-level encoded state in our work, the probabilities are given by $p_{\mathrm{G}} = 4.1 \cdot 10^{-5}$, $p_{\mathrm{A}} = 0.05$ and $p_{\mathrm{D}} = 0.87$ and the laser repetition rate was $f_{\mathrm{R}} = 400$ MHz.

Appendix E: Towards universal one-way quantum computing

The distribution of cluster states is an essential prerequisite for several quantum networking applications based on universal one-way quantum computing, including blind photonic quantum computing, where quantum information is shared between spatially separated clients and servers to enable secure and anonymous quantum processing [8]. Our platform could be extended to implement universal one-way computing on distributed cluster states, permitting e.g., the implementation of a distributed, blind version of Grover's algorithm. Today, such network protocols have only been demonstrated over very short distances (~ 10 m), which are typically impractical for real-world applications [1, 2, 9]. Our platform is capable of closing this gap and could enable the demonstration of distributed algorithms with up to 5 multi-level encoded qubits per photon across spatially separated locations – a regime that currently is not addressable in quantum networking.

In this section, we discuss how independent local measurements on qubits in arbitrary bases and classical feed forward operations – the essential ingredients of universal one-way computing [10] – could be implemented in our platform.

1. Local measurements of qubits in arbitrary bases

In order to measure a given multi-level encoded qubit in an arbitrary local basis, an appropriate unitary must be applied prior to the photon measurement. Typically, such a unitary basis transformation required linear optical elements such as a beam splitters and phase shifters. A reconfigurable time-bin beam splitter likewise allows the implementation of any unitary operation on a given multi-level time-bin encoded qubits. This is because we can access both the splitting ratio as well as the phase of the beam splitter. Utilizing the chirped pulse modulation, we can implement the beam splitter operation, i.e. transforming the time-bin encoded qubits (basis states $|0\rangle, |1\rangle$) of a given qubit into two superposition states given by

Table 2. **Scaling of generation, transmission, and detection rates for $2n$-qubit states in multi-level and multi-photon encoding.** In both cases, resources are assumed to be probabilistic photon sources and universal linear optics.

Rate	Multi-level encoding	Multi-photon encoding	benefit
$R_{\mathrm{G}}(n)$	$f_{\mathrm{R}} \cdot p_{\mathrm{G}} \cdot (1/2)^n$	$f_{\mathrm{R}} \cdot (p_{\mathrm{G}})^n (1/2)^{n-1}$	$break - even$
$R_{\mathrm{T}}(n,l)$	$e^{-2\alpha l}$	$e^{-2n\alpha l}$	$\mathcal{O}(e^{(n-2)\alpha l)})$
$R_{\mathrm{P}}(n)$	$(p_A)^{2n} \cdot (p_D)^2$	$(p_A \cdot p_D)^{2n}$	$break - even$

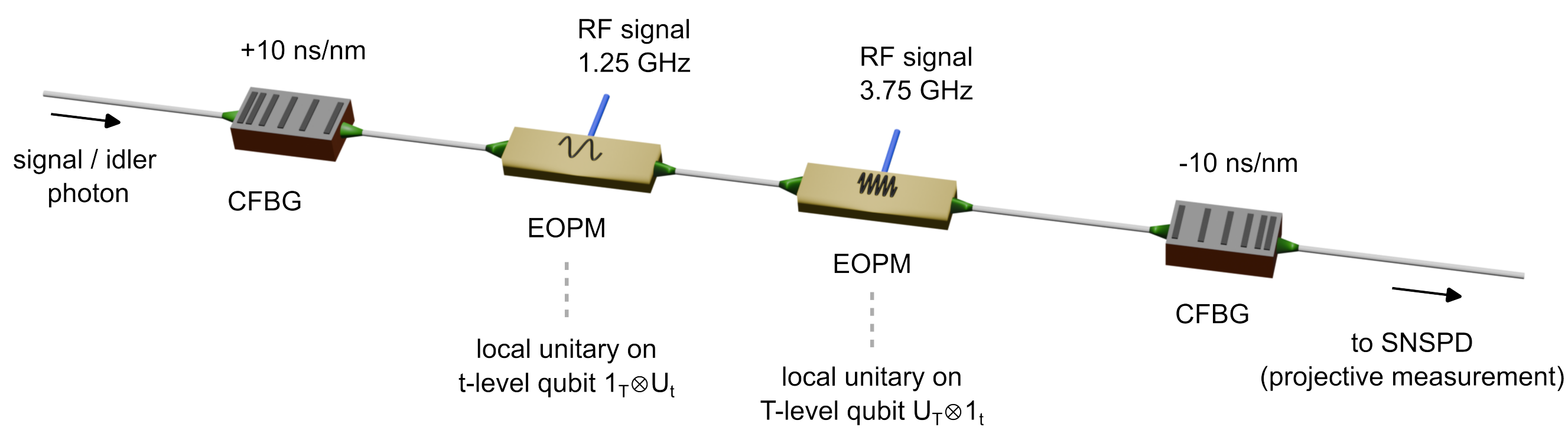

Figure S5. **Proposed CPM configuration for the implementation of one-way computing on two qubits encoded per photon.** To access both qubits, two time-bin beam splitters are required. Chirped fiber Bragg gratings (CFBGs) of opposite dispersion compensate, thus simply a second electro-optic phase modulator (EOPM) has to be placed into the CPM setup. By adjusting the amplitude and (relative) phase of the $\omega/(2\pi) = 1.25$ GHz RF signal on the first EOPM, any local unitary $1_T \otimes U_t$ can be implemented on the T-level qubit. Similarly, by adjusting the amplitude and (relative) phase of the $\omega/(2\pi) = 3.75$ GHz RF signal on the second EOPM, any local unitary $U_T \otimes 1_t$ can be implemented on the t-level qubit. The resulting operation on both qubits is given by $U_T \otimes U_t$. Projective measurement of both qubits in this combined basis is achieved by routing the photon onto a superconducting nanowire single photon detector (SNSPD).

$$|\alpha_\pm\rangle = \frac{1}{N}(J_0(g)|0\rangle \pm J_1(g)e^{i\alpha}|1\rangle), \quad \text{(S1)}$$

where $N = (|J_0|^2 + |J_1|^2)$ is the normalization constant and J_0, J_1 denote the Bessel functions of first kind. By tuning the modulation depth g and the phase α that is given by the relative delay between the RF signal and the arrival time of the photons, we can access any (superposition) state on the Bloch sphere, thus facilitating local measurements of qubits in arbitrary bases.

For the reconstruction of the cluster state witness and the one-way computing primitives in our experiment, we already implemented measurements in three different bases. For measurements in the Z-basis (basis states $|0\rangle, |1\rangle$) we set the modulation depth $g = 0$ and α can be set to an arbitrary value. For measurements in the X-basis (basis states $|+\rangle, |-\rangle$) we set the modulation depth to $g = 1.4342$ and the phase to $\alpha = 0$. For measurements in the XY-plane on the Bloch sphere (basis states $|\alpha_+\rangle = (|0\rangle + e^{i\alpha}\ |1\rangle)/\sqrt{2},\ |\alpha_-\rangle = (|0\rangle - e^{i\alpha}|1\rangle)/\sqrt{2})$ we have set $g = 1.4342$ and α corresponding to the equatorial rotation angle. While the measurements in the X-basis and Z-basis are required to reconstruct the witness, the measurements in the XY-basis were used to certify one-way quantum operations.

2. Measurement of multiple qubits per photons

To implement universal one-way quantum computing operations, it is required to perform local measurements not only on a single qubit of the cluster state in an arbitrary basis, but also on multiple qubits. Importantly, this does not require multiple projective measurements per photon, but we can perform a projective measurement on each qubit simultaneously with a single detection event. To implement this, one time-bin beam splitter is required per qubit acting on the relevant time-bin spacing to coherently interfere the basis states of this qubit. To this end, a photon carrying multiple qubits must be passed through a sequence of the time-bin beam splitter. Each time-bin beam splitter requires one electro-optic phase modulators (EOPM) and two chirped fiber Bragg grat-

ings (CFBGs) of opposite dispersion. However, CFBGs with positive and negative dispersion compensate, thus the implementation effectively only requires a sequence of EOPMs placed inbetween the CFBGs, see Fig. S5. In our experimental scenario, where two qubits are encoded per photon, this results in two cascaded EOPMs, where the first one is driven at a frequency of $\omega/(2\pi) = 1.25$ GHz to superimpose time-bins separated by 100 ps (arbitrary local basis measurement on t-level qubit) followed by a second one which is driven at $\omega/(2\pi) = 3.75$ GHz to superimpose time-bin separated by 300 ps (arbitrary local basis measurement on T-level qubit).

3. Classical feed-forward

Classical feed forward requires that the measurement outcome of a local qubit determines the basis measurements on subsequent qubits. This mechanism can be implemented either activley, i.e. the next measurement is delayed until the measurement apparatus has been reconfigured based on the previous measurement outcome, or passively, i.e. the next measurement is already intrinsically preconditioned on previous measurement outcomes. To achieve classical feed-forward in our system, both approaches need to be implemented. Specifically, feed-forward between qubits encoded into different photons needs to be implemented activley. This can be achieved leveraging the relative delay between the photons and the fast reconfigurability of the CPM, nativley permitting a fast change in measurement basis, e.g. via a FPGA. In contrast, feed-forward between qubits encoded into the same photon can be realized passively by varying the relative timing between time-bin levels and the applied RF signal, as this temporal adjustment can provide a pre-programming of the time-bin beam splitter phase settings.

Appendix F: High-capacity quantum processing via spectral multiplexing

Time-bin encoded qubits require a specific volume in the time-frequency domain, fundamentally constrained by their time-bandwidth product. In our encoding scheme, each qubit occupies approximately 25 GHz (corresponding to 0.2 nm) of spectral bandwidth and 200 ps of temporal bandwidth. Given that a chirped fiber Bragg grating with a dispersion of 10 ns/nm stretches the time-bins to 2 ns, the maximum repetition rate without introducing time-bin crosstalk is 500 MHz. Assuming an available optical bandwidth of 5 THz (covering the full C-band), the resulting maximum processable information rate reaches 100 GigaQubits/s.

Appendix G: The (non-)ideal time-bin beam splitter

1. Experimental imperfections

The time-bin beam splitter implemented in our CPM setup is limited by coherent scattering of photons into undesired modes, which prevents us from implementing deterministic measurements in unbiased operations. In contrast, an ideal time-bin beam splitter coherently superimposed two temporal input modes and maps them onto two temporal output modes. In our implementation, the intrinsic efficiency depends on the modulation strength g applied to the phase modulator in the CPM setup and is given by

$$\eta = |J_0(g)|^2 + |J_1(g)|^2 \tag{S1}$$

where J_0, J_1 are the Bessel functions of first kind. This results in an intrinsic efficiency of $\eta(0) = 1.0$ for measurement in the Z-basis (no beam splitting) and $\eta(1.4342) = 0.601$ for measurements in the XY-plane (symmetric beam splitting). In the evaluation of our two-photon cluster state experiments, we normalization each projections with respect to the total counts in the measurement basis (i.e., $ZZZZ$, $ZZXX$, $XXZZ$) to account for this imperfection.

2. Numerical investigation

To implement measurements in unbiased bases and boost the intrinsic efficiency of the time-bin beam splitter arbitrarily close to $\eta = 1.0$, multiple stages of CPMs and EOPMs can be cascaded. We closely follow related concept for arbitrary universal temporal transformations [11, 12] to numerically investigate the implementation of an ideal time-bin beam splitter by a CPM-EOPM-CPM cascade, see Fig. S6. In particular we optimize the phase parameters of the EOPM as well as the modulation depths and relative phases of the CPMs up to the third harmonic. We optimize the two-mode transformation V of the CPM-EOPM-CPM cascade with respect to the process fidelity

$$F = \frac{tr(V^\dagger W)tr(W^\dagger V)}{tr(V^\dagger V)tr(W^\dagger W)} \tag{S2}$$

to target the unitary two-mode transformation W of an ideal time-bin beam splitter (i.e., a Hadamard transformation) for a time-bin spacing of 100 ps. The optimization procedure (Broyden–Fletcher–Goldfarb–Shannon) converged to a configuration with an intrinsic efficency of $\eta = 0.977$ at a process fidelity of 99.72%, see Fig. S7. This procedure also takes the spectral walk-off into account. It is known that in order to reach $\eta = 1$, multi-tone modulation with an infinite amount of driving

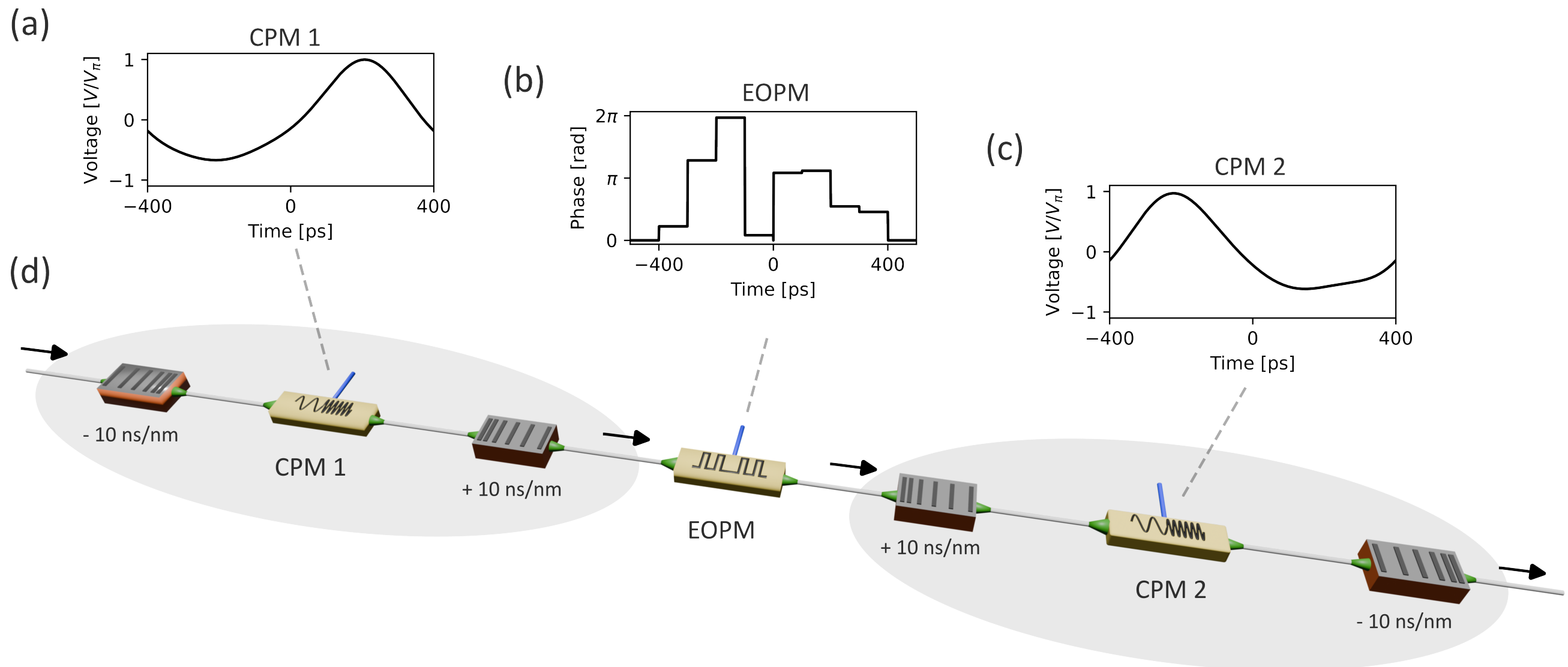

Figure S6. **Proposed implementation of a quasi-ideal time-bin beam splitter for** 100 **ps time-bin spacing** (a) The first CPM is driven by a modulation signal composed of three tones (1.25 GHz, 2.5 GHz and 3.75 GHz).(b) The EOPM applies phases to each time-bin after the first CPM. (c) The second CPM is again driven by a modulation signal composed of the fundamental and two harmonics. (d) Schematic illustration of the CPM-EOPM-CPM cascade. Areas shaded in gray represent a CPM.

tones is required [13].

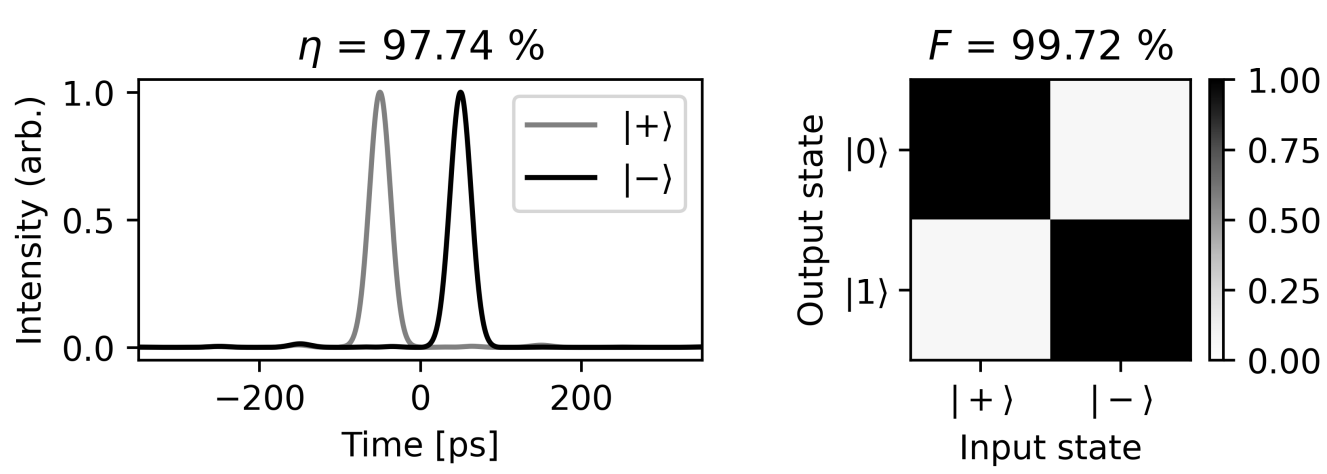

Figure S7. **Numerical results for the quasi-ideal time-bin beam splitter for 100 ps time-bin spacing.** The temporal output distribution (left) indicated strongly suppressed scattering into undesired modes resulting in an intrinsic efficiency of 97.74%. The (unitary) transformation resembles the unitary operation of a symmetric beam splitter with a process fidelity of 99.72 %.

To achieve an advantage beyond break-even in total efficiency (i.e. intrinsic efficiency and losses), the quasi-ideal time-bin beam splitter from the CPM-EOPM-CPM cascade would need to outperform the non-ideal time-bin beam splitter form the CPM setup. For simplicity, the losses per component are assumed to be identical for EOPMs and CFBGs and denoted by l. For the non-ideal time-bin beam splitter we consider three components and an intrinsic efficiency of $\eta_1 = 0.601$ and for the quasi-ideal CPM–EOPM–CPM cascade, we consider seven components and an intrinsic efficiency of $\eta_2 = 0.977$. To reach beyond break-even the condition is $\eta_1 l^3 < \eta_2 l^7$, which results in $l < 0.886$ for the transmission probability per component. This corresponds to a maximum of 0.52 dB insertion loss per component to operate the quasi-ideal time-bin beam splitter in a regime beyond break-even.

Appendix H: Literature comparison

We have compared several aspects of our work with representative literature. In Table 3 chirped pulse modulation is compared with other platforms for time-bin manipulation relying on post-selection. Table 4 compares our approach to cluster-state generation and transmission with other time-frequency encoded cluster-state implementations. Table 5 compares our transmission distance with that achieved in other experiments involving the transmission of time-bin encoded states.

Table 3. **Comparison of different architectures for (post-selected) manipulation of time-bin photons.** Note that this is not a complete list. Further relevant references can be found for example in [14, 15]. Coupling losses for photonic circuits are excluded from the budget.

Architecture	Ref.	Spacing	Loss	Visibility	Intrinsic efficiency	Reconfiguration time	Stabilization
Fiber Interferometer	[16]	100 ns	4.4 dB	97.1 %	50%	$\sim$ 100 ns	Optical feedback
Fiber Interferometer	[17]	4 ns	1.1 dB	88.4 %	50%	$\sim$ 100 μs	Piezo/PID
Photonic circuit (SiPh)	[18]	20 ns	4.5 dB	88.4 %	50%	$\sim$ 1 ms	Thermal/PID
Photonic circuit (SiPh)	[19]	64 ps	12.5 dB	91.1 %	50%	–	Thermal/PID
Photonic circuit (LN)	[20]	220 ps	2.1 dB	78.1 %	50%	–	Thermal/PID
CPM (This work)	–	100 ps	8.1 dB	96.8 %	60.1 %	2 ns	RF Reference

Table 4. **Comparison of different photonic cluster states encoded in time and frequency.** Neither discrete-variable (DV), nor continuous-variable (CV) cluster states have been transmitted over relevant distances (> 1 km) of optical fiber.

Cluster state	Reference	State space	Fiber-based	Transmission distance
Time/Frequency-bin (DV)	[21]	2 photons, 4 qudits	Yes	–
Time/Frequency-bin (DV)	[22]	2 photons, 6 qubits	Yes	–
2D-Time-multiplexed (CV)	[23]	30000 Qumodes	Yes	–
1D-Time-multiplexed (CV)	[24]	10000 Qumodes	Yes	–
2D-Time-multiplexed (CV)	[25]	6200 Qumodes	Yes	–
3D-Time-multiplexed (CV)	[26]	20 Qumodes	No	–
Time-bin (DV)	This work	2 photons, 4 qubits	Yes	29.5 km

Table 5. **Comparison of experiments demonstrating the fiber transmission of quantum states over practically relevant distances.** Here, the distance is reported as an absolute value and does not take the distribution topology into account.

Transmitted state	Ref.	Encoding	Distance	Deployed
Bell state	[27]	Polarization	248 km	Yes
Bell state	[28]	Polarization	192 km	Yes
Bell state	[29]	Polarization	155 km	Yes
Bell state	[30]	Time-bin	300 km	No
Bell state	[31]	Polarization	404 km	No
Bell state	[32]	Time-bin	60 km	No
Bell state	[33]	Frequency-bin	24 km	No
Bell state	[34]	Frequency-bin	25 km	No
High-dimensional	[35]	Time-bin	100 km	No
Squeezed state	[36]	–	40 km	No
Squeezed state	[37]	–	1 km	Yes
Squeezed state	[38]	OAM	1 km	No
Squeezed state	[39]	–	6 km	No
GHZ state	[7]	Polarization	50 km	No
Cluster state	This work	Time-bin	29.5 km	partially

1. Main, D. *et al.* Distributed quantum computing across an optical network link. *Nature* 1–6 (2025).
2. Polacchi, B. *et al.* Multi-client distributed blind quantum computation with the qline architecture. *Nature Communications* **14**, 7743 (2023).
3. Nielsen, M. A. & Chuang, I. L. *Quantum computation and quantum information* (Cambridge: Cambridge University Press, 2010).
4. Clements, W. R., Humphreys, P. C., Metcalf, B. J., Kolthammer, W. S. & Walmsley, I. A. Optimal design for universal multiport interferometers. *Optica* **3**, 1460–1465 (2016).
5. Rudin, B. *et al.* High-performance mode-locking with up to 50 ghz repetition rate from integrable vecsels. In *2006 Conference on Lasers and Electro-Optics and 2006 Quantum Electronics and Laser Science Conference*, 1–2 (IEEE, 2006).
6. Proximion AB. High dispersion compensation module datasheet. (2025-05). at `https://www.proximion.com/hubfs/bu-pr/datasheet/dispersion-compensation/DCM-HDC_Datasheet.pdf` URL.
7. Proietti, M. *et al.* Experimental quantum conference key agreement. *Science Advances* **7**, eabe0395 (2021).
8. Broadbent, A., Fitzsimons, J. & Kashefi, E. Universal blind quantum computation. In *2009 50th annual IEEE symposium on foundations of computer science*, 517–526 (2009).
9. Barz, S. *et al.* Demonstration of blind quantum computing. *science* **335**, 303–308 (2012).
10. Raussendorf, R. & Briegel, H. J. A one-way quantum computer. *Physical Review Letters* **86**, 5188 (2001).
11. Lukens, J. M., Islam, N. T., Lim, C. C. W. & Gauthier, D. J. Reconfigurable generation and measurement of mutually unbiased bases for time-bin qudits. *Applied Physics Letters* **112** (2018).
12. Ashby, J. *et al.* Temporal mode transformations by sequential time and frequency phase modulation for applications in quantum information science. *Optics Express* **28**, 38376–38389 (2020).
13. Lu, H.-H. *et al.* Electro-optic frequency beam splitters and tritters for high-fidelity photonic quantum information processing. *Physical Review Letters* **120**, 030502 (2018).
14. Xavier, G. B., Larsson, J.-Å., Villoresi, P., Vallone, G. & Cabello, A. Energy-time and time-bin entanglement: past, present and future. *npj Quantum Information* **11**, 129 (2025).
15. Montaut, N. *et al.* Progress in integrated and fiber optics for time-bin based quantum information processing. *Advanced optical technologies* **14**, 1560084 (2025).
16. Monika, M. *et al.* Quantum state processing through controllable synthetic temporal photonic lattices. *Nature Photonics* **19**, 95–100 (2025).
17. Roztocki, P. *et al.* Arbitrary phase access for stable fiber interferometers. *Laser & Photonics Reviews* **15**, 2000524 (2021).
18. Xiong, C. *et al.* Compact and reconfigurable silicon nitride time-bin entanglement circuit. *Optica* **2**, 724–727 (2015).
19. Yu, H. *et al.* Quantum key distribution implemented with d-level time-bin entangled photons. *Nature Communications* **16**, 171 (2025).
20. Finco, G. *et al.* Time-bin entangled bell state generation and tomography on thin-film lithium niobate. *npj Quantum Information* **10**, 135 (2024).
21. Reimer, C. *et al.* High-dimensional one-way quantum processing implemented on d-level cluster states. *Nature Physics* **15**, 148–153 (2019).
22. Gaeta, A., Blau, M., Ji, X. & Lipson, M. All-photonic 2xn cluster states in frequency, time and polarization (2025).
23. Larsen, M. V., Guo, X., Breum, C. R., Neergaard-Nielsen, J. S. & Andersen, U. L. Deterministic generation of a two-dimensional cluster state. *Science* **366**, 369–372 (2019).
24. Yokoyama, S. *et al.* Ultra-large-scale continuous-variable cluster states multiplexed in the time domain. *Nature Photonics* **7**, 982–986 (2013).
25. Asavanant, W. *et al.* Generation of time-domain-multiplexed two-dimensional cluster state. *Science* **366**, 373–376 (2019).
26. Roh, C., Gwak, G., Yoon, Y.-D. & Ra, Y.-S. Generation of three-dimensional cluster entangled state. *Nature Photonics* **19**, 526–532 (2025).
27. Neumann, S. P., Buchner, A., Bulla, L., Bohmann, M. & Ursin, R. Continuous entanglement distribution over a transnational 248 km fiber link. *Nature Communications* **13**, 6134 (2022).
28. Wengerowsky, S. *et al.* Passively stable distribution of polarisation entanglement over 192 km of deployed optical fibre. *npj Quantum Information* **6**, 5 (2020).
29. Du, J. *et al.* Entanglement distribution over 155 km metropolitan fiber using a cmos-compatible silicon chip. *Newton* **1** (2025).
30. Inagaki, T., Matsuda, N., Tadanaga, O., Asobe, M. & Takesue, H. Entanglement distribution over 300 km of fiber. *Optics express* **21**, 23241–23249 (2013).
31. Zhuang, S.-C. *et al.* Ultrabright entanglement based quantum key distribution over a 404 km optical fiber. *Physical Review Letters* **134**, 230801 (2025).
32. Kim, J.-H., Chae, J.-W., Jeong, Y.-C. & Kim, Y.-H. Quantum communication with time-bin entanglement over a wavelength-multiplexed fiber network. *APL Photonics* **7** (2022).
33. Kues, M. *et al.* On-chip generation of high-dimensional entangled quantum states and their coherent control. *Nature* **546**, 622–626 (2017).
34. Khodadad Kashi, A. & Kues, M. Frequency-bin-encoded entanglement-based quantum key distribution in a reconfigurable frequency-multiplexed network. *Light: Science & Applications* **14**, 49 (2025).
35. Ikuta, T. & Takesue, H. Four-dimensional entanglement distribution over 100 km. *Scientific reports* **8**, 817 (2018).
36. Suleiman, I. *et al.* 40 km fiber transmission of squeezed light measured with a real local oscillator. *Quantum Science & Technology* **7**, 045003 (2022).
37. Verclas, S., Darsow-Fromm, C., Tohermes, B. & Schnabel, R. Fiber distribution of phase-stabilized ghz-bandwidth squeezed vacuum states of light between two buildings. *Optica Quantum* **4**, 1–6 (2025).
38. Ren, Y., Wang, X., Lv, Y., Bacco, D. & Jing, J. Distribution of multiplexed continuous-variable entanglement

for quantum networks. *Laser & Photonics Reviews* **16**, 2100586 (2022).

39. Huo, M. *et al.* Deterministic quantum teleportation through fiber channels. *Science advances* **4**, eaas9401 (2018).
